# De novo targeting to the cytoplasmic and luminal side of bacterial microcompartments

**DOI:** 10.1038/s41467-018-05922-x

**Published:** 2018-08-24

**Authors:** Matthew J. Lee, Judith Mantell, Ian R. Brown, Jordan M. Fletcher, Paul Verkade, Richard W. Pickersgill, Derek N. Woolfson, Stefanie Frank, Martin J. Warren

**Affiliations:** 1 Industrial Biotechnology Centre, School of Biosciences, University of Kent, Canterbury, CT2 7NJ UK; 2 School of Biochemistry, University of Bristol, Medical Sciences Building, University Walk, Bristol, BS8 1TD UK; 3 Wolfson Bioimaging Facility, Medical Sciences Building, University Walk, Bristol, BS8 1TD UK; 4 School of Chemistry, University of Bristol, Cantock’s Close, Bristol, BS8 1TS UK; 5 BrisSynBio, Life Sciences Building, Tyndall Avenue, Bristol, BS8 1TQ UK; 6 School of Biological and Chemical Sciences, Queen Mary University of London, Mile End Road, London, E1 4NS UK; 7 Department of Biochemical Engineering, University College London, Bernard Katz Building, Gordon Street, London, WC1E 6BT UK

## Abstract

Bacterial microcompartments, BMCs, are proteinaceous organelles that encase a specific metabolic pathway within a semi-permeable protein shell. Short encapsulation peptides can direct cargo proteins to the lumen of the compartments. However, the fusion of such peptides to non-native proteins does not guarantee encapsulation and often causes aggregation. Here, we report an approach for targeting recombinant proteins to BMCs that utilizes specific de novo coiled-coil protein–protein interactions. Attachment of one coiled-coil module to PduA (a component of the BMC shell) allows targeting of a fluorescent protein fused to a cognate coiled-coil partner. This interaction takes place on the outer surface of the BMC. The redesign of PduA to generate an N-terminus on the luminal side of the BMC results in intact compartments to which proteins can still be targeted via the designed coiled-coil system. This study provides a strategy to display proteins on the surface or within the lumen of the BMCs.

## Abstract (web summary)

Bacterial microcompartments (BMCs) are protein-bound organelles encapsulating segments of metabolic pathways. Here the authors utilize specific de novo coiled-coil protein-protein interactions to display proteins on the outer or inner surface of BMCs.

## Introduction

Although once thought to lack subcellular organization, prokaryotes utilize a number of mechanisms to systematically arrange cellular enzymes to both enhance metabolic pathways and also to protect cellular components from potentially damaging intermediates. Re-engineering heterologous metabolic pathways with a higher level of organization has become of increasing interest to synthetic biologists looking to maximize yields from engineered pathways^1^. Such mechanisms include protein-based scaffolds, DNA scaffolds, protein aggregation, and compartmentalization^2–6^. However, in some eubacteria, nature has evolved small protein-based organelles called bacterial microcompartments (BMCs) that contain a specific metabolic pathway^7^. These BMCs, which are found in around 20% of bacteria, consist of a protein shell that encases the enzymes involved in particular metabolic pathways^8–11^. A recent computational study has revealed that the primary function of BMCs is to enhance luminal intermediate concentrations leading to an enhancement in metabolic flux^12^. In addition, there is convincing evidence that BMCs protect cells from the toxicity associated with high aldehyde concentrations^13,14^.

BMCs can be broadly divided into two subclasses, anabolic carboxysomes and catabolic metabolosomes^7,15^. Carboxysomes encase the enzymes carbonic anhydrase and ribulose-1,5-bisphosphate carboxylase/oxygenase (RuBisCO) leading to high levels of the substrate CO_2_ to overcome the poor catalytic activity of RuBisCO^16,17^. Of the catabolic metabolosomes, the organelle associated with 1,2-propanediol utilization (Pdu) is, to date, the best characterized^18–20^. This structure, ~150 nm in diameter, houses the enzymes required for the metabolism of 1,2-propanediol (1,2-PD) via the toxic intermediate propionaldehyde to form 1-propanol and propionyl-CoA^19,21^. The genetic information encoding this metabolic module is housed on a 23-gene operon, the so-called *pdu* operon^19^. Seven genes encode for eight proteins that form the shell of the BMC (PduA, B, B′, J, K, N, U, T), which either form hexameric (BMC-H), trimeric (BMC-T) or pentameric (BMC-P) disks that assemble to form the shell^22–25^. Indeed, a recent structural study has revealed the organization of these proteins in a recombinant BMC structure, whereby the shell proteins are orientated with their concave side facing outwards^26^. Although this clearly indicates a specific orientation for the shell proteins within a recombinant BMC, there is also evidence to support the opposite, concave-in, arrangement in native systems^27,28^.

The biotechnological potential of BMCs was first realized through the expression of the entire *pdu* operon in *E. coli* resulting in the formation of functional metabolosomes^29^. Further work showed that through the expression of a minimal set of BMC shell proteins (PduA, B, B′, J, K, N and U) empty BMCs could be readily produced and isolated^30^. Proteins can be targeted to such empty BMCs by fusion to short targeting sequences, namely, D18 and P18, that are naturally found on the N-terminus of the proteins PduD and PduP, respectively, which are integral components of the Pdu metabolosome^27,31,32^. These targeting peptides have been used to show the targeting of fluorescent proteins to BMCs, and, more recently, they have been utilized to construct an in vivo ethanol bioreactor^4^. For the latter, the enzymes pyruvate decarboxylase and alcohol dehydrogenase have been targeted to recombinant BMCs by fusion to the D18 and P18 targeting peptides. The resulting strains are able to produce more ethanol than strains producing the enzymes cytoplasmically, demonstrating that compartmentalization is beneficial to this recombinant pathway. Other work has shown that it is possible to utilize targeting peptides from different BMCs to target proteins to 1,2-PD utilization BMCs. This promiscuity suggests a relatively basic mechanism of targeting^33^. Indeed, sequence analysis reveals a conserved hydrophobic motif among BMC systems and subsequent work by Jakobson et al. shows that it is possible to design targeting peptides de novo based on this hydrophobic motif^33,34^.

Recent work has shown that fusion of the D18 and P18 targeting peptides to proteins of an engineered 1,2-PD synthesis pathway results in poor levels of protein encapsulation and variable levels of protein aggregation^3^. Nonetheless, when co-produced together these large enzyme aggregates produce significantly more product than the untagged cytoplasmic variants. Therefore, such active enzyme aggregates may provide an alternative strategy to enhance metabolic flux. However, aggregation may be problematic if the full potential of BMCs is to be realized and therefore alternative targeting mechanisms may facilitate the further development of BMC technology. Towards this goal researchers have recently used protein-interaction domains such as PDZ, GBD and SH3 to target fluorescent proteins to recombinant BMCs in the host *C. glutamicum*^35^.

Here, we describe an altogether different strategy for targeting recombinant proteins to BMCs, utilizing de novo designed coiled-coils^36,37^. By attaching one half of an obligate coiled-coil heterodimer to the N-terminal, cytoplasm-facing region of PduA, we demonstrate that it is possible to target to it with cargo that has been tagged with the cognate coiled-coil partner. To target cargo to the luminal side of the BMC, PduA was engineered so that its N*-*terminus is luminal facing. This permutated version of PduA still forms BMCs when coproduced with the other shell proteins and the use of the heterodimeric coiled-coil peptide system then allows for internalization of tagged cargo.

## Results

### Construction of coiled-coil labeled BMCs

Previously, it has been shown that heterologous proteins can be targeted to BMCs by fusion to short targeting peptides such as D18 or P18, however such fusions often result in protein aggregation^3,31,32^. The de novo coiled-coil peptides CC-Di-A and CC-Di-B have been designed to interact specifically with each other and to avoid self-association^36,37^. Using these peptides, we demonstrated recently that filaments composed of a CC-Di-B-PduA* fusion can be targeted with fusions of CC-Di-A to fluorescent proteins such as Citrine and mCherry^2^. The PduA hexamers within these filaments are also orientated with the concave side facing outwards into the cytoplasm^2,38^. Therefore, we wondered if the CC-Di-A/CC-Di-B (CC-Di-AB) cognate pairing could also be used to target proteins to intact BMCs. To investigate this, we generated a range of coiled-coil-PduA fusions with the aim of incorporating the fused PduA protein into BMCs within *E. coli*. To achieve this the coiled-coil peptides were fused to the N-terminus of PduA via a glycine/serine-based linker (GS Linker) but separated by a hexa-histidine tag and a thrombin cleavage site (Supplementary Table 1). This led to the construction of synthetic genes encoding for CC-Di-A-PduA and CC-Di-B-PduA. The N-terminus of wild-type PduA is located on the concave side of the hexamer and thus in assembled recombinant BMCs faces the cellular cytoplasm^26,38^. Hence, targeting to the N-terminus of PduA is presumed to append cargo proteins to the outer surface of the organelle.

In other constructs, the sequence for a fluorescent protein (Citrine) was incorporated between the thrombin cleavage site and PduA to allow for the production of fluorescently labeled, coiled-coil tagged, BMC shells (Supplementary Table 1). This gave CC-Di-A-Citrine-PduA and CC-Di-B-Citrine-PduA. A control construct, called C-PduA, containing the GS Linker, hexa-histidine tag and thrombin cleavage site, but without a coiled-coil sequence, was also made. The separate synthetic genes for these modified PduA variants were incorporated into a plasmid containing the remaining genes for the other shell proteins (PduB, B′, J, K, N, U) required to form empty BMCs. In this way a total of six plasmids encoding CC-Di-A-PduA-U, CC-Di-B-PduA-U, C-PduA-U, CC-Di-A-Citrine-PduA-U, CC-Di-B-Citrine-PduA-U, and C-Citrine-PduA-U were transformed individually into *E. coli* BL21 * (DE3) cells (Supplementary Table 1). The resulting strains were grown, induced and analyzed by transmission electron microscopy (TEM) after fixation, embedding, thin sectioning and staining. TEM analysis revealed the presence of BMC structures within thin sections of cells from all six strains that were grown (Supplementary Figure 1).

The BMCs from the six strains were purified as outlined in methods and were subjected to SDS-PAGE analysis. All of the purified BMCs produced banding patterns typical of a BMC, where the major bands visualized included PduB, PduB′, PduJ, PduK and PduU. From the strains harboring CC-Di-B-PduA and CC-Di-B-Citrine-PduA, the modified PduAs were readily discernable (Supplementary Figures 2, 3, 4). In contrast, purifications of BMCs isolated from strains producing CC-Di-A-PduA or C-PduA did not contain a band of the expected size for PduA suggesting that these variants are not incorporated into the BMC shell, although it should be noted that these fusion proteins are not as well produced by *E. coli* in comparison to CC-Di-B tagged variants (Supplementary Figures 4–8).

**Fig. 1:**
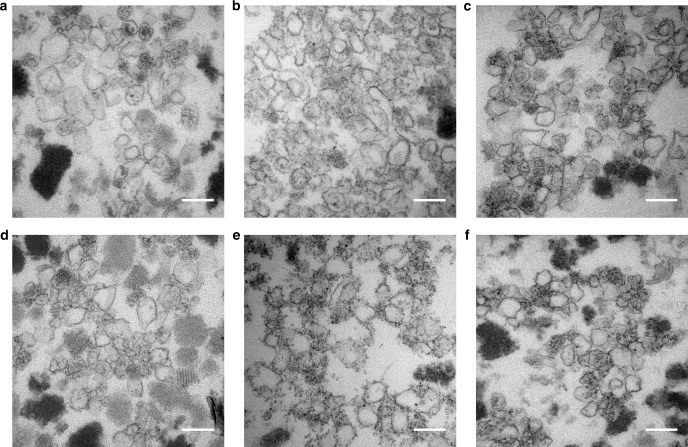
TEM analysis of resin embedded, thin-sectioned, purified BMCs. BMCs extracted from *E. coli* BL21 * (DE3) cells expressing the following modified PduA proteins. **a** CC-Di-A-PduA-U. **b** CC-Di-B-PduA-U. **c** C-PduA-U. **d** CC-Di-A-Citrine-PduA-U. **e** CC-Di-B-Citrine-PduA-U. **f** C-Citrine-PduA-U. Scale bars show 0.2 µm

To gain a better understanding of the structural characteristics of the modified compartments, the purified BMCs were fixed, embedded, thin sectioned and stained. TEM analysis revealed closed compartments in all samples, which had outline shapes and dimensions similar to native empty Pdu BMCs that had been prepared and imaged in the same way. The sectioning of BMCs randomly embedded in resin means that a range of sizes and shapes will be observed and hence this approach does not provide detailed analyses of the compartments (Fig. 1, Supplementary Figures 9, 10). However, the micrographs show a number of structures within the purified samples, which appear to be broken BMCs. It is possible that these BMCs may have been damaged during the purification process, which involves high-speed centrifugation. Lipids and protein aggregates are also visualized in purified samples, a consequence of the relatively crude purification process.

Next, fluorescently labeled BMC variants were examined by confocal microscopy to confirm that the modified PduAs are incorporated into the shells of the BMCs. The strain producing the CC-Di-B-Citrine-PduA-U BMCs contained fluorescent puncta, indicating that CC-Di-B-Citrine-PduA is indeed incorporated into the BMC (Supplementary Figures 11 (B) and 12 (D)). However, strains producing CC-Di-A-Citrine-PduA-U and C-Citrine-PduA-U do not show any such puncta, suggesting that CC-Di-A-Citrine-PduA and C-Citrine-PduA are not incorporated into BMCs. These results are consistent with the SDS-PAGE and western blot data, where the PduA variants are not observed in the latter (Supplementary Figures 11 (A and C) and 12 (A and G)).

**Fig. 2:**
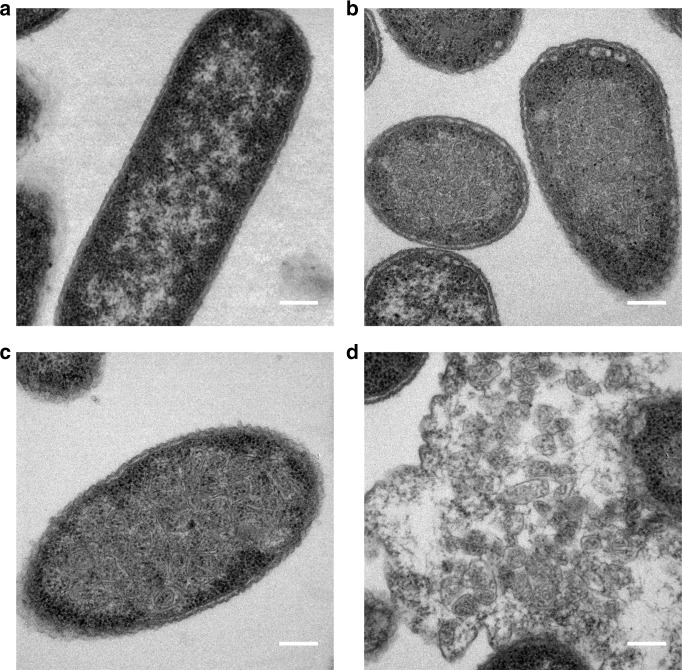
Minimal requirements for BMC shell formation. TEM analyses of resin embedded, thin sectioned, *E. coli* BL21 * (DE3) cells (**a**) control strain transformed with empty vector (**b**) producing the shell proteins PduA, B, B′, J, K, N and U (PduA-U) (**c**, **d**) producing the shell proteins PduB, B′, J, K, N and U (PduB-U). **c** In vivo, and **d** from lysed cells. Scale bars show 0.2 µm

Although PduA is a major component of the Pdu BMC it is not essential for BMC formation in *S. enterica*^14^. This explains why BMCs are observed in samples even though PduA variants are not incorporated into the structure. To confirm that PduA is also not essential for our recombinant BMC system, we constructed a plasmid containing the genes required for BMC formation but lacking *pduA*; i.e., with *pduB, B’*′, *J, K, N* and *U*. TEM analysis of the strain expressing PduB-U revealed correctly formed BMCs within the cytoplasm of *E. coli* (Fig. 2 and Supplementary Figure 13).

### Targeting to BMCs via coiled-coil interactions

As mentioned previously, one problem with the natural targeting peptides, D18 and P18, is that, when fused to some cargo proteins, they can elicit protein aggregation, presumably as a result of the amphipathic nature of these peptides^3,27^. To investigate if similar protein aggregation occurs with the coiled-coil peptides CC-Di-A and CC-Di-B, the peptides were fused separately to the fluorescent protein mCherry. Cells expressing the corresponding synthetic genes in *E. coli* were visualized by confocal microscopy and TEM. With both the control (C-mCherry) and the CC-Di-A-mCherry fusion strains a dispersed cytoplasmic fluorescence was observed, which is indicative of a soluble protein (Supplementary Figures 14 (A and C) and 15 (A and G). However, expression of CC-Di-B-mCherry resulted in localized areas of increased protein density as evidenced by fluorescent puncta in the cells and areas of electron density in TEM micrographs, suggesting that some self-association of the CC-Di-B-based fusion had taken place. However, it should be noted that amorphous protein aggregates as observed with fusions to native BMC targeting peptides were not visible by TEM (Supplementary Figures 14 (B) and 15 (D, E and F)).

**Fig. 3:**
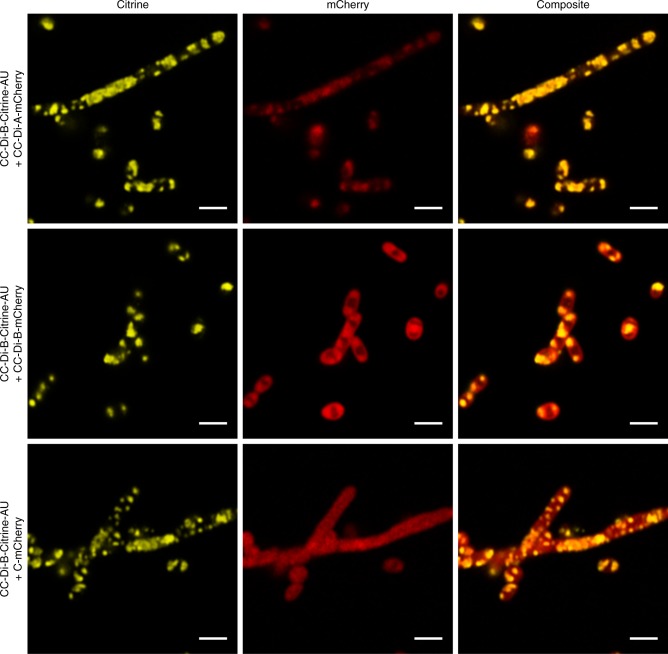
Localization of fluorescent proteins to coiled-coil labeled BMCs. Confocal analysis of *E. coli* BL21 * (DE3) cells showing Citrine, mCherry and composite fluorescence signals for cells expressing CC-Di-B-Citrine-PduA-U with CC-Di-A-mCherry, CC-Di-B-mCherry or C-mCherry. Scale bars show 2 µm

The CC-Di-B-PduA fusion protein appears to be incorporated into empty BMCs when co-produced with PduB, B′, J, K, N and U. As the CC-Di-B is attached to the N-terminus of PduA, and as the N-terminus of PduA, in the hexamer, is on the concave side of the hexamer, evidence suggests that within the BMC the CC-Di-B peptide will be facing into the cytoplasm of the cell^26,38^. To test if the coiled-coil peptides were accessible to their cognate partner peptide, plasmids encoding CC-Di-B-Citrine-tagged BMCs were co-expressed with CC-Di-A-tagged mCherry. Confocal microscopy revealed co-localized fluorescent puncta within the bacterial cells, indicative of an association between the two-tagged components. By contrast, in a control strain producing the same CC-Di-B-Citrine-labeled BMCs and C-mCherry, the mCherry signal remained cytoplasmic indicating no interaction with the BMC (Fig. 3 and Supplementary Figure 16). Expression of CC-Di-B-tagged BMCs with CC-Di-B-tagged mCherry also resulted in co-localization of the florescence, but in this case the areas of colocalization were large, polar and more reminiscent of the areas of increased protein density that are observed in the strain expressing only CC-Di-B-mCherry.

### Redesign of PduA with a lumen-facing N terminus

The BMC shell protein PduU contains the same characteristic BMC domain as PduA, however, it is circularly permuted; i.e., the protein secondary structure elements are identical but are arranged in a different order, which results in the N- and C*-* termini being located on opposing faces of the hexamer^39,40^. However, the low occupancy of PduU in recombinant BMC shells precludes its effective use in incorporating recombinant proteins into the BMC lumen via coiled-coil interactions^21^. Therefore, we aimed to circularly permute the abundant BMC shell protein PduA to make it analogous to PduU; i.e., to relocate the N- and C- termini to the convex side of the hexameric disk whilst retaining the characteristic BMC domain architecture^41^.

**Fig. 4:**
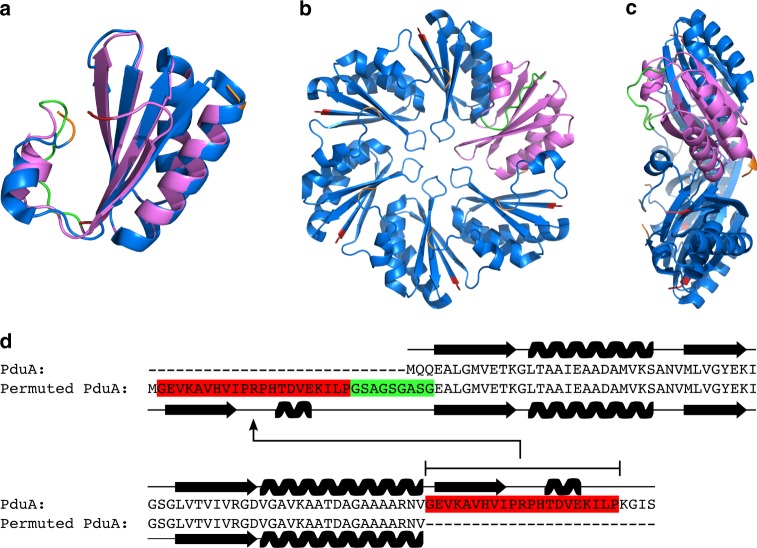
Circular permutation of the shell protein PduA. **a** Computationally generated model of our permuted PduA (PduA^P^) generated with Phyre2^45^ (magenta) overlaid with the native structure (blue) with the flexible linker joining the native *N-* and *C-* termini highlighted in green. **b**, **c** Architecture of the native PduA hexamer 3NGK [http://dx.doi.org/10.2210/pdb3ngk/pdb] (blue) illustrating the permuted variant PduA^P^ (magenta). N*-* and C*-* termini are highlighted in red and orange respectively in (**a**, **b**) and **c**. **d** Methodology for creating a circularly permuted PduA with the transplanted region highlighted in red and the linker region in green

This redesign of PduA involved moving part of the C*-*terminus to the N-terminus. Based on the X-ray crystal structure of PduA^42^, Val-68 was chosen as the site for the permutation; it is exposed on the surface of the hexamer, and we hypothesized that permutation of the segment after this residue should have minimal effect on the structure of the hexamer. To achieve this, the C-terminal region of PduA (GEVKAVHVIPRPHTDVEKILP) was grafted onto the N-terminus, with a flexible linker (GSAGSGASG) used to connect the wildtype N*-* and C*-* termini (Fig. 4). A recent study employed a similar strategy, however, in this case it was found that the permutation resulted in a change in the oligomeric state of the protein from a hexamer to a pentamer, a change that resulted in its self-assembly into a 13 nm nanocage^43^.

DNA encoding the circularly permuted PduA (PduA^P^) was synthesized, the gene cloned into a plasmid and transformed into *E. coli* BL21 * (DE3) cells. The resultant strain was grown, and the cells harvested, fixed, dehydrated, embedded and thin sectioned. Subsequent analysis by TEM revealed the presence of 3 nm thick ribbon-like structures within the cell, which often appear stacked together in parallel (Supplementary Figure 17 (A–F)). Tomography of these cells reveals that the ribbons extend down the full length of the section (Supplementary Movie 1), consistent with the ribbons representing an edge-on view of protein sheets that have been cut transversely. Therefore, we conclude that the ribbon-like structures represent protein sheets formed from hexamers of PduA^P^. Such sheet-like structures would not form if PduA^P^ existed as a pentamer as has been observed with a variant permuted PduA that had been constructed in a slightly different way in a previous study^43^.

The synthetic gene encoding the PduA^P^ was next combined in a plasmid with the other genes required to form an empty BMC shell (*pduB, B*′*, J, K, N, U*) generating the construct pLysS-PduA^P^-U. This plasmid was transformed into BL21 * (DE3) competent cells and analyzed by TEM as described above.

**Fig. 5:**
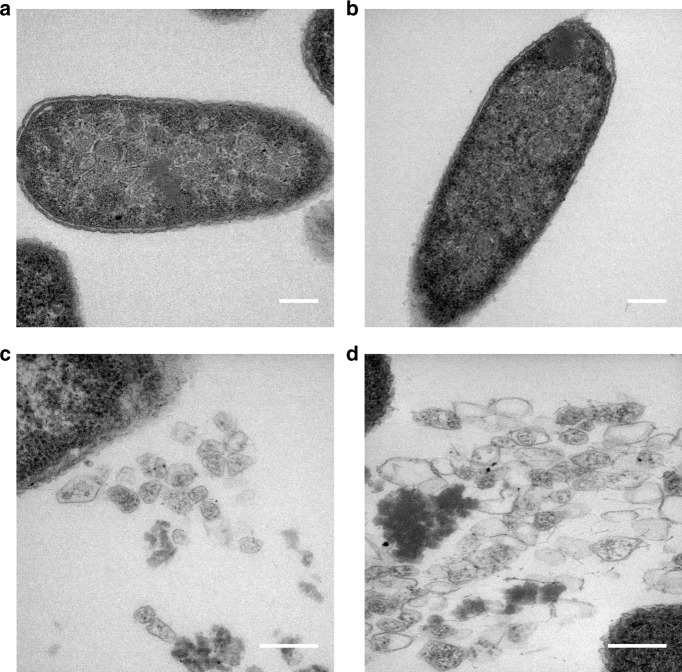
Expression of circularly permuted BMCs. TEM analyses of resin embedded, thin sectioned, *E. coli* BL21 * (DE3) cells producing wild type PduA BMCs and circularly permuted PduA^P^ BMCs. **a**, **c** PduA-U in-vivo and isolated (**b**, **d**) PduA^P^-U. Scale bars show 0.2 µm

TEM revealed the presence of BMCs in vivo that were structurally similar to empty BMCs made from wildtype proteins (PduA-U), showing that the permutation does not have a noticeable effect on the structure (Fig. 5A, B; Supplementary Figure 18). In addition, BMCs are also observed free in the resin, presumably as a result of some cell lysis, and are morphologically similar to their wildtype PduA-containing counterparts (Fig. 5C, D; Supplementary Figure 18), providing further evidence that the circular permutation does not affect the structure of BMCs. However, attempts to purify BMCs containing PduA^P^, using the previously well-established extraction procedures, failed. Presumably, this is because incorporation of PduA^P^ into BMCs changes their solubility characteristics. To demonstrate that PduA^P^ is incorporated into the BMCs in vivo, a fluorescent version of PduA^P^ was constructed integrating the CC-Di-B-Citrine module onto its N-terminus. Confocal microscopy of the strain producing this CC-Di-B-Citrine-PduA^P^-U complex revealed florescent puncta, consistent with this fusion being successfully incorporated into the BMC shell (Supplementary Figures 19 B and 20 D). In contrast, strains expressing CC-Di-A-Citrine-PduA^P^-U or C-Citrine-PduA^P^-U did not have a punctate fluorescent phenotype suggesting that these variants are not incorporated into the shell of the BMC as seen with the non-permuted variants (Supplementary Figures 19 (A and C) and 20 (A and G)).

### Targeting to BMCs containing PduA^P^

**Fig. 6:**
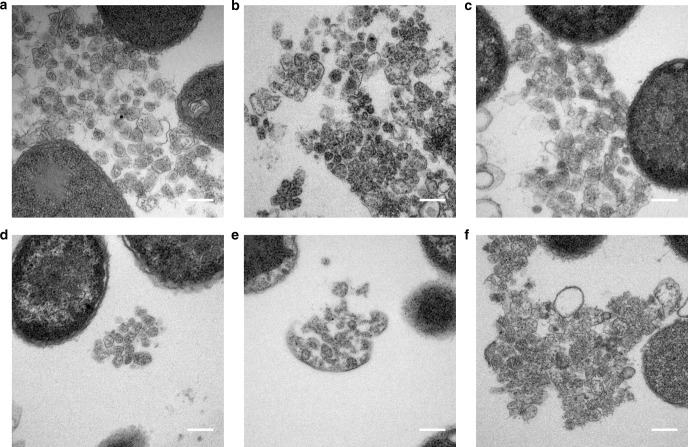
TEM analysis of coiled-coil tagged circularly permuted BMCs. BMCs tagged with coiled-peptides from lysed *E. coli* BL21 * (DE3) cells expressing the following modified PduA proteins. **a** CC-Di-A-PduA^P^-U. **b** CC-Di-B-PduA^P^-U. **c** C-PduA^P^-U. **d** CC-Di-A-Citrine-PduA^P^-U. **e** CC-Di-B-Citrine-PduA^P^-U. **f** C-Citrine-PduA^P^-U. Scale bars show 0.2 µm

To investigate targeting to PduA^P^, we utilized the same CC-Di-AB system used with PduA^36,37^. Addition of CC-Di-B to PduA^P^, and in the presence of PduB-U, did not disrupt BMC formation (Supplementary Figure 21). However, we noted that some malformed BMCs were present in these strains, but only to a similar level as observed for the unmodified empty shell construct (PduA-U) (Supplementary Figures 10 and 21). BMCs that appeared to have been released through cell lysis offered better visualization of the structures, and these were morphologically similar to the unmodified control (PduA-U) and to permuted BMCs lacking the coiled-coil sequence (Figs. 5, 6 and Supplementary Figure 22).

**Fig. 7:**
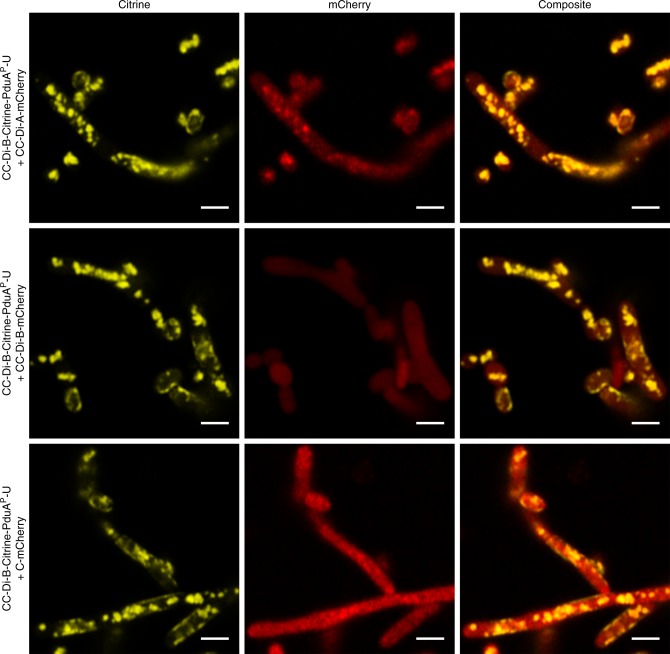
Localization of fluorescent proteins to circularly permuted coiled-coil labeled BMCs. Confocal analysis of *E. coli* BL21 * (DE3) cells showing Citrine, mCherry and composite fluorescence signals for cells expressing CC-Di-B-Citrine-PduA^P^-U in conjunction with CC-Di-A-mCherry, CC-Di-B-mCherry or C-mCherry. Scale bars show 2 µm

Next, we sought to target cargo protein to the coiled-coil tagged PduA^P^ within the BMC. Strains were transformed with a plasmid encoding CC-Di-B-Citrine-PduA^P^-U and a second plasmid producing the fluorescent protein reporter (mCherry) tagged with either CC-Di-A, CC-Di-B or lacking a coiled-coil sequence (C-mCherry). Strains were grown and analyzed by confocal fluorescence microscopy (Fig. 7 and Supplementary Figure 23). Co-localized fluorescence signals were apparent in the strain expressing CC-Di-B-Citrine-PduA^P^-U and CC-Di-A-mCherry, although it must be noted that a high amount of cytoplasmic CC-Di-A-mCherry was also present, suggesting that not all of the protein is targeted to the BMC (lumen). A wholly cytoplasmic mCherry signal was observed in the strain producing CC-Di-B-Citrine-PduA^P^-U together with untagged mCherry (C-mCherry) (Fig. 7). Together, these results provide strong evidence that it is possible to utilize de novo designed coiled-coil peptides to target to the luminal side of BMCs.

## Discussion

Previously, we have shown that a de novo designed and highly specific coiled-coil peptide pair, termed CC-Di-A and CC-Di-B, can be used to target proteins to filamentous tubules formed in the cytoplasm of *E. coli*. These tubules, which we collectively call a cytoscaffold, are formed by the overproduction of a soluble form of the natural BMC protein PduA, termed PduA*^2^. With this system, we find that CC-Di-B-PduA* filaments bind protein cargoes tagged with CC-Di-A, results that are consistent with the N-terminus of PduA* facing the cellular cytoplasm.

Therefore, and as detailed herein, we wondered if the same coiled-coil system could be used to direct cargo to an empty recombinant BMC within *E. coli*. Such BMCs are formed from the coordinated production of multiple shell proteins, PduA-U. To achieve our goal, we investigated the effect of adding CC-Di-A or CC-Di-B to the N-terminus of wildtype PduA and co-producing these variants with the remaining shell proteins. We find that only the CC-Di-B-PduA variant incorporates into the recombinant BMCs. It appears that CC-Di-A makes the PduA unstable, which is also what we observe for tagged versions of PduA*^2^.

By coproducing CC-Di-B-PduA with PduB-U, intact BMCs are observed by TEM. Similar BMCs are also observed for a CC-Di-B-Citrine-PduA version produced with PduB-U. In this case, the presence of BMCs was additionally confirmed by confocal microscopy, which reveals fluorescent puncta within the cellular cytoplasm. The ability of the CC-Di-A peptide to bind to the CC-Di-B-PduA variants was demonstrated in vivo with a CC-Di-A-mCherry fusion protein where co-production of the latter with the BMC containing the CC-Di-B-Citrine-PduA gives co-localized puncta within cells. In these cases, on the basis of the X-ray crystal structure of PduA and further recent experimental evidence including the structure determination of a recombinant BMC as well as regiospecific imaging of PduA filaments and targeted proteolysis of PduA fusions within BMCs, we suggest that the mCherry is being directed to the outer, cytoplasm-facing side of these recombinant BMCs^26,38^.

However, it should be noted that there still remains some ambiguity over the orientation of shell proteins in native BMCs with some evidence suggesting interactions between luminal enzymes and the concave faces of shell proteins, indicative of the concave face being luminal^27,28^. Conversely, in recombinant systems, including the system used here, evidence points towards the concave face being cytoplasmic^26,38^. For the purposes of this research we are working on the assumption that the N- and C-terminii of PduA are located on the concave outward-facing side of the protein.

Therefore, and following on from this, we speculated the coiled-coil peptides could be used to target to the luminal side of the BMC through the redesign of PduA to relocate the N-terminus to the convex side of the hexamer. To achieve this, we permutated PduA after residue 68 to maintain its overall structure by moving the N- and C*-* termini to the opposite side of the molecule. This permutated form of PduA, PduA^P^, forms recombinant BMCs in vivo when co-produced with PduB-U, although these could not be recovered for detailed in vitro analysis, suggesting that the PduA^P^ changes the physical properties of the BMC in some way.

Nonetheless, both CC-Di-B and CC-Di-B-Citrine can be fused to PduA^P^ to render competent recombinant BMCs. Indeed, the co-production of CC-Di-B-Citrine-PduA^P^ with PduB-U gives fluorescent puncta within the cells, consistent with the incorporation of PduA^P^ into the BMC. Again, targeting to the CC-Di-B-PduA^P^-containing BMCs was demonstrated with a CC-Di-A-mCherry fusion and the co-localization of Citrine and mCherry signals. These results are consistent with our objective of incorporating cargo protein within the lumen of the BMC using coiled-coil technology.

In summary, we have developed methods for the localization of proteins to both the outside and the inside of BMCs. Targeting to the outside could be used to display proteins on a comparatively large protein framework, for example, in the generation of antibodies. The internal accommodation of proteins can be used to engineer new metabolic pathways within the BMC; such endowed BMCs could be used as delivery vehicles. Through these approaches we have shown that the field of cellular and metabolic engineering depends not only on the implementation of natural architectures but also on de novo design, in order to achieve maximal functionality.

## Methods

### Cloning of constructs

DNA was synthesized by Eurofins Genomics, sequence information is shown in Supplementary Data 1, 2 and 3. Plasmids were constructed as outlined in Supplementary Table 1 and Supplementary Data 4 with primers listed in Supplementary Table 2. JM109 competent cells were used for all cloning steps (Supplementary Table 3).

### Growth of strains producing BMCs

BL21* (DE3) competent cells were transformed with appropriate plasmids. LB (50 mL) supplemented with ampicillin (100 mg/L) and chloramphenicol (34 mg/L) in baffled flasks was inoculated from an overnight starter culture. Cells were grown at 37 °C with shaking to an OD_600_ ∼ 0.4, protein production was induced by addition of IPTG to a final concentration of 400 µM. Cultures were incubated for 2 h at 19 °C with shaking for confocal and TEM analysis or overnight with shaking for TEM analysis and purification.

### Cell lysis

Cells were harvested by centrifugation at 2683 × *g*, a one gram wet cell pellet was resuspended in 20 mL Yeast Protein Extraction Reagent (Y-PER) supplemented with protease Inhibitor Cocktail Tablets, EDTA-Free and 500 Units benzonase nuclease and incubated for 3 h at room temperature with gentle shaking.

### Purification of CC-Di-B tagged BMCs

BMCs were pelleted from the lysate by centrifugation for 5 min at 11,300 × *g*, the pellet was resuspended in 2 mL 20 mM Tris-HCl pH 8, 20 mM NaCl. The suspension was centrifuged at 4 °C for 5 min at 11,000 × *g*. The BMC containing pellet was resuspended in 2 mL 20 mM Tris-HCl pH 8 and centrifuged for 5 min at 11,000 × *g*, the BMC containing supernatant was collected and the NaCl concentration was increased to 80 mM. The suspension was centrifuged as above. The pellet was resuspended in an appropriate volume of 20 mM Tris-HCl pH 8 for analysis.

### Purification of CC-Di-A tagged and untagged BMCs

BMCs were pelleted from the lysate by centrifugation for 5 min at 11,300 × *g*, the pellet was resuspended in 2 mL of 20 mM Tris-HCl, pH 8, containing 20 mM NaCl. The suspension was centrifuged at 4 °C for 5 min at 11,000 × *g*. The supernatant, containing microcompartments was collected; the NaCl concentration was increased to 80 mM. The suspension was centrifuged at 4 °C for 5 min at 11,000 × *g*, the resulting pellet contained BMCs.

### Western blot analysis

Following transfer and blocking nitrocellulose membranes were incubated in primary antibody (mouse anti-His (Sigma-Aldrich; catalog #H1029; diluted 1:3000 in PBS)) for 2 h. Membranes were washed three times in PBS then equilibrated for 10 min in 50 mM Tris-HCl pH 7.5, 150 mM NaCl. Membranes were then incubated in a secondary antibody coupled to alkaline phosphatase (Anti-Mouse IgG (H + L), AP Conjugate (Promega; catalog #S3721; diluted 1:5000 in 50 mM Tris-HCl pH 7.5, 150 mM NaCl)). Bands were visualized by incubation in substrate 5-bromo-4-chloro-3-indolyl phosphate/nitro blue tetrazolium (BCIP/NBT).

### Embedding of samples for TEM analysis

Following growth and induction of protein expression cells were harvested by centrifugation at 3000 × *g*. For embedding of BMCs the final pellet from the purification was used in place of a cell pellet. Pellets were fixed in 2 ml 2.5% glutaraldehyde in 100 mM sodium cacodylate buffer pH 7.2 (CAB) and incubated for 2 h with gentle rotating. Cells/BMCs were pelleted by centrifugation at 6000 × *g* for 2 min and were washed twice with 100 mM CAB for 10 min. Cells were stained with 1% osmium tetroxide in 100 mM CAB for 2 h and then washed twice with ddH_2_O prior to dehydration. Dehydration was carried out by incubation in an ethanol gradient, 50% EtOH for 10 min, 70% EtOH overnight, 90% EtOH for 10 min, 100% dry EtOH for 10 min 3 times. Pellets were washed twice with propylene oxide for 15 min. Samples were embedded by first washing in 1 ml of a 1:1 mix of propylene oxide and Agar LV Resin for 30 min with rotation and then by suspension in freshly prepared 100% Agar LV resin twice for 1.5 h. The pellet was re-suspended in fresh resin and transferred to a 1 ml Beem embedding capsule (size 00) and subsequently centrifuged for 5 min at 3000 × *g* to concentrate the cells to the tip of the mold. Polymerization was achieved by incubation at 60 °C for 20 h.

### Sectioning and visualization of samples

Following embedding samples were ultra-thin sectioned on a RMC MT-XL ultra-microtome with a diamond knife (diatome 45°) thin sections (60–70 nm) were collected on un-coated 300 mesh copper grids. Grids were first stained by incubation in 4.5% uranyl acetate in 1% acetic acid for 45 min followed by washing for 10 s in a stream of ddH_2_O to remove any excess stain. Grids were subsequently stained with Reynolds lead citrate for 7 min followed by washing in a stream of ddH_2_O.

Sections were observed and photographed using a JEOL-1230 transmission electron microscope equipped with either a Gatan multiscan or Gatan OneView digital camera operated at an accelerating voltage of 80 kV.

### Confocal imaging

Following growth and induction of protein expression 1 ml of cells were harvested by centrifugation at 3000 × *g*. The cell pellet was washed in PBS three times then fixed in 2% (w/v) formaldehyde in PBS 15 min, cells were subsequently washed a further 3 times in PBS. Cells (10 μL) were pipetted onto a 1.5 thickness coverslip (0.17 ± 0.005 mm) before being inverted onto a drop of ProLong Gold antifade mountant (Life Technologies) on a glass slide. Samples were cured for 24 h in the dark prior to imaging.

Images were acquired on a Zeiss LSM 880 with Airyscan system. Excitation light (514 nm for Citrine or 561 nm for mCherry) was provided by an argon laser (514 nm) or HeNe Laser (561). Images were acquired using a 100 × 1.46 NA oil immersion objective lens.

### Tomography

Sections (250 nm) were cut from existing blocks, and 15 nm gold fiducials (Aurion, TomoSol solution) were applied to both sides of the sections. Sections were imaged using a Tecnai 20 TEM (FEI, the Netherlands) operated at 200 kV and tilt series images were acquired between –66° to + 66° with 1.5° (above 45°) and 2° increments (below 45°). Images were acquired on a 4k by 4k FEI Eagle camera with a pixel size of 1.38 nm. The resulting tomograms were reconstructed and combined using IMOD software^44^.

## Electronic supplementary material

Supplementary Information

Descriptions of Additional Supplementary Files

Supplementary Movie 1

Supplementary Data 1

Supplementary Data 2

Supplementary Data 3

Supplementary Data 4

## Data Availability

All data, including images and clones, are available upon request from the authors.
