# Supplementary material for: De novo targeting to the cytoplasmic and luminal side of bacterial microcompartments
Source: Nat Commun. 2018 Aug 24;9:3413. doi: 10.1038/s41467-018-05922-x (PMC6109187; doi:10.1038/s41467-018-05922-x)
Supplement: Supplementary file 1 — Supplementary Information [file 41467_2018_5922_MOESM1_ESM.docx]

***De novo* targeting to the cytoplasmic and luminal side of bacterial microcompartments**

Lee *et al.*


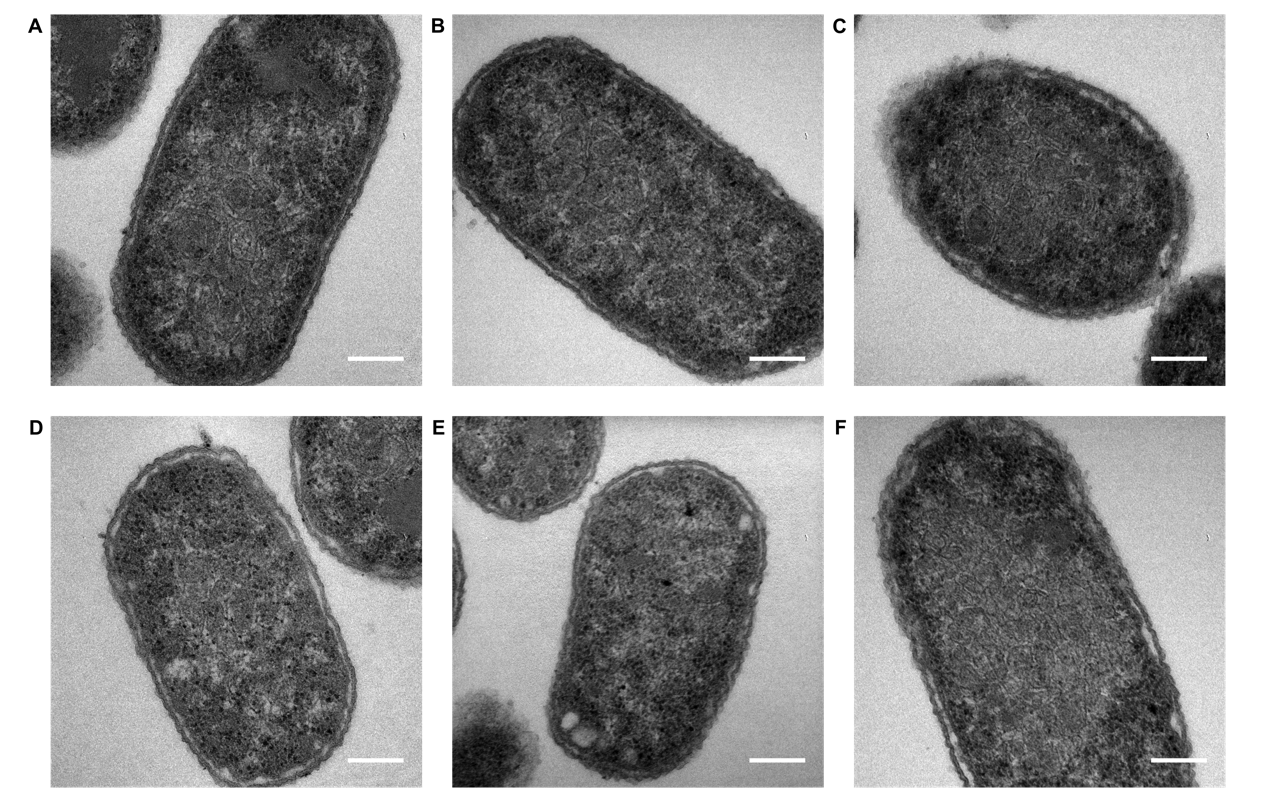


**Supplementary Figure 1. *In-vivo* expression of coiled-coil labelled BMCs.** TEM analysis of *E. coli* BL21 * (DE3) cells expressing: (**A**) CC-Di-A-PduA-U (**B**) CC-Di-B-PduA-U (**C**) C-PduA-U (**D**) CC-Di-A-Citrine-PduA-U (**E**) CC-Di-B-Citrine-PduA-U (**F**) C-Citrine-PduA-U. Scale bars show 0.2 µm.


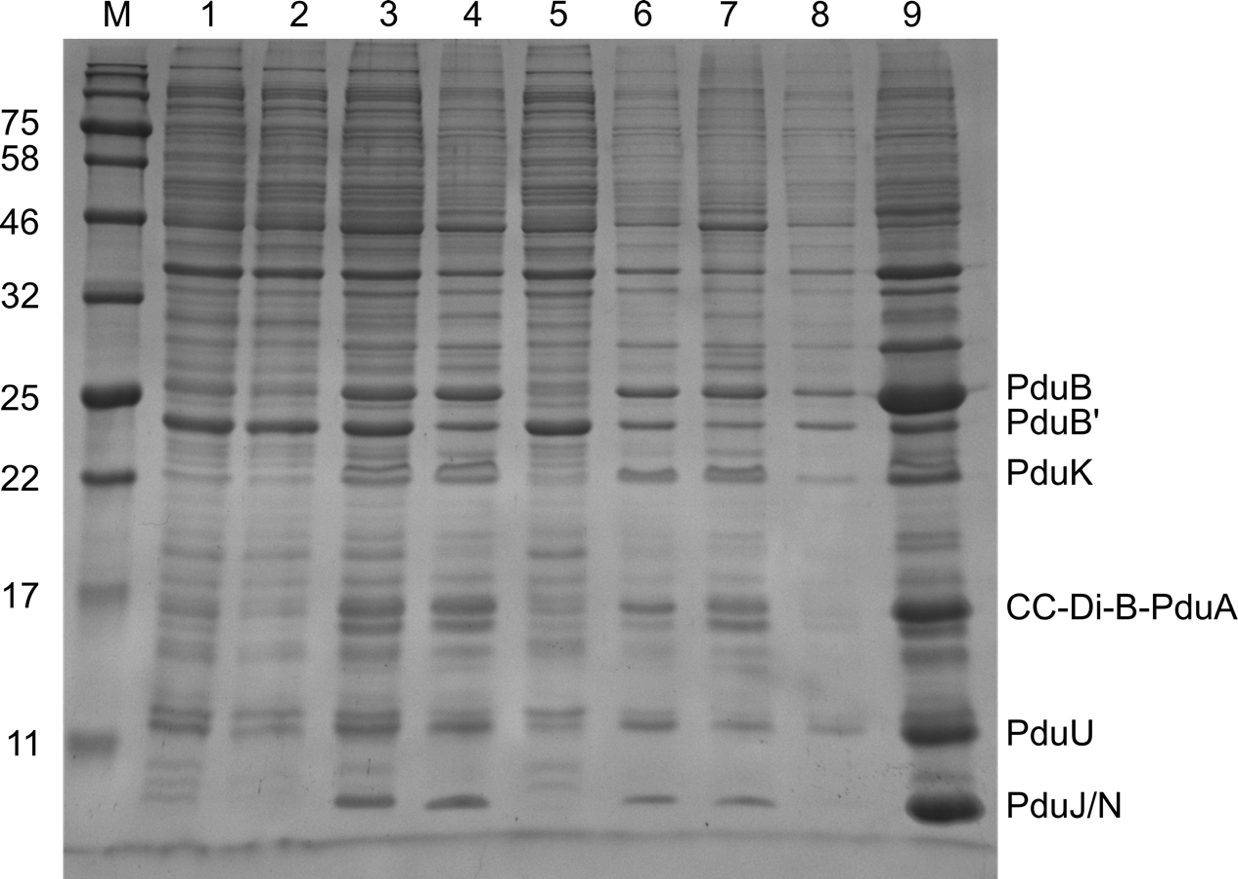


**Supplementary Figure 2.** **SDS-PAGE analysis of CC-Di-B labeled BMC purification in comparison to a molecular weight marker (M) (kDa).** Lane 1 – lysate, lane 2 – supernatant after centrifugation at 11,300 x g, lane 3 – pellet after centrifugation (resuspended in 20 mM Tris-HCl, pH 8, containing 20 mM NaCl), lane 4 – pellet after centrifugation at 11,000 x g (resuspended in 20 mM Tris-HCl, pH 8, containing 20 mM NaCl), lane 5 – supernatant after centrifugation, lane 6 - supernatant after centrifugation of resuspended pellet, lane 7 – pellet after centrifugation of resuspended pellet, lane 8 - supernatant after centrifugation, lane 9 pellet after centrifugation in 80 mM NaCl, resuspended in 20 mM Tris-HCl pH 8. Sample volumes of 2 µL were loaded in lanes 1 and 2, whilst lanes 3 to 9 were loaded with 5 μL sample.


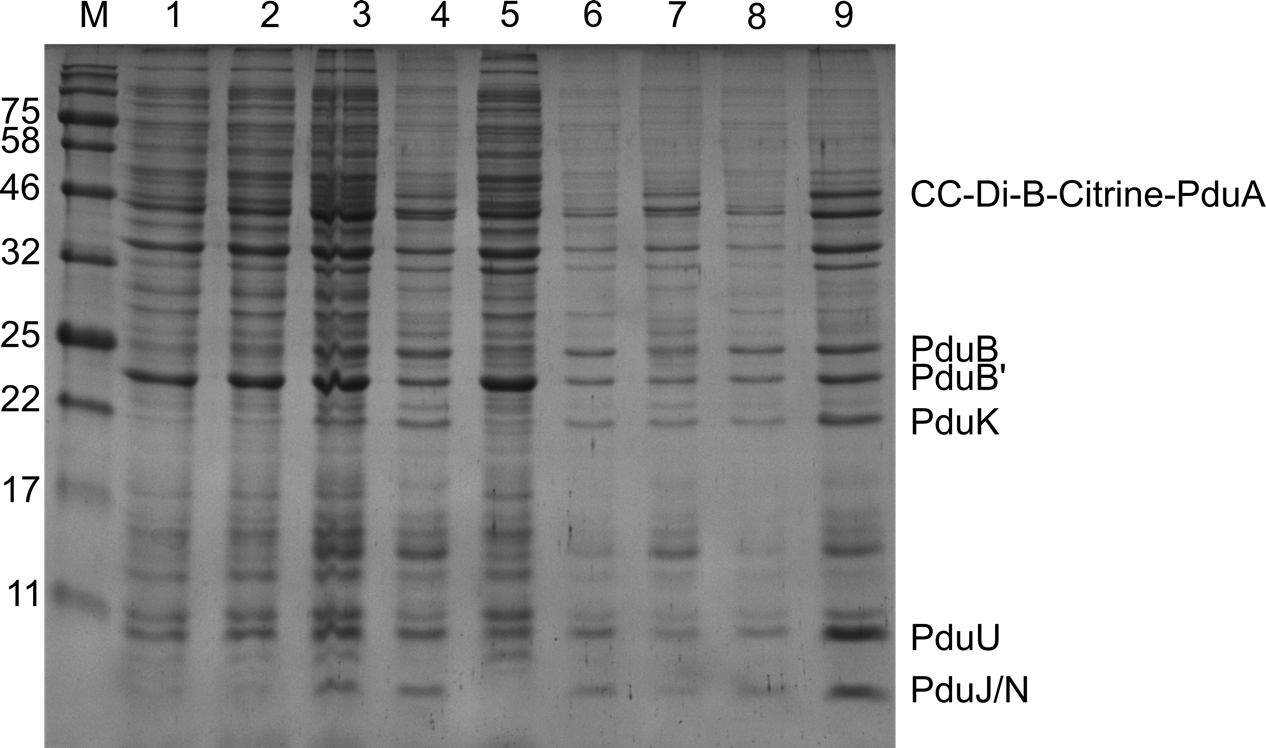


**Supplementary Figure 3.** **SDS-PAGE analysis of CC-Di-B-Citrine labeled BMC purification in comparison to a molecular weight marker (M) (kDa).** Lane 1 – lysate, lane 2 – supernatant after centrifugation at 11,300 x g, lane 3 – pellet after centrifugation (resuspended in 20 mM Tris-HCl, pH 8.0, containing 20 mM NaCl), lane 4 – pellet after centrifugation at 11,000 x g (resuspended in 20 mM Tris-HCl, pH 8.0, containing 20 mM NaCl), lane 5 – supernatant after centrifugation, lane 6 - supernatant after centrifugation of resuspended pellet, lane 7 – pellet after centrifugation of resuspended pellet, lane 8 - supernatant after centrifugation, lane 9 pellet after centrifugation in 80 mM NaCl, resuspended in 20 mM Tris-HCl, pH 8. Sample volumes of 2 µL were loaded in lanes 1 and 2, whilst lanes 3 to 9 were loaded with 5 μL sample.


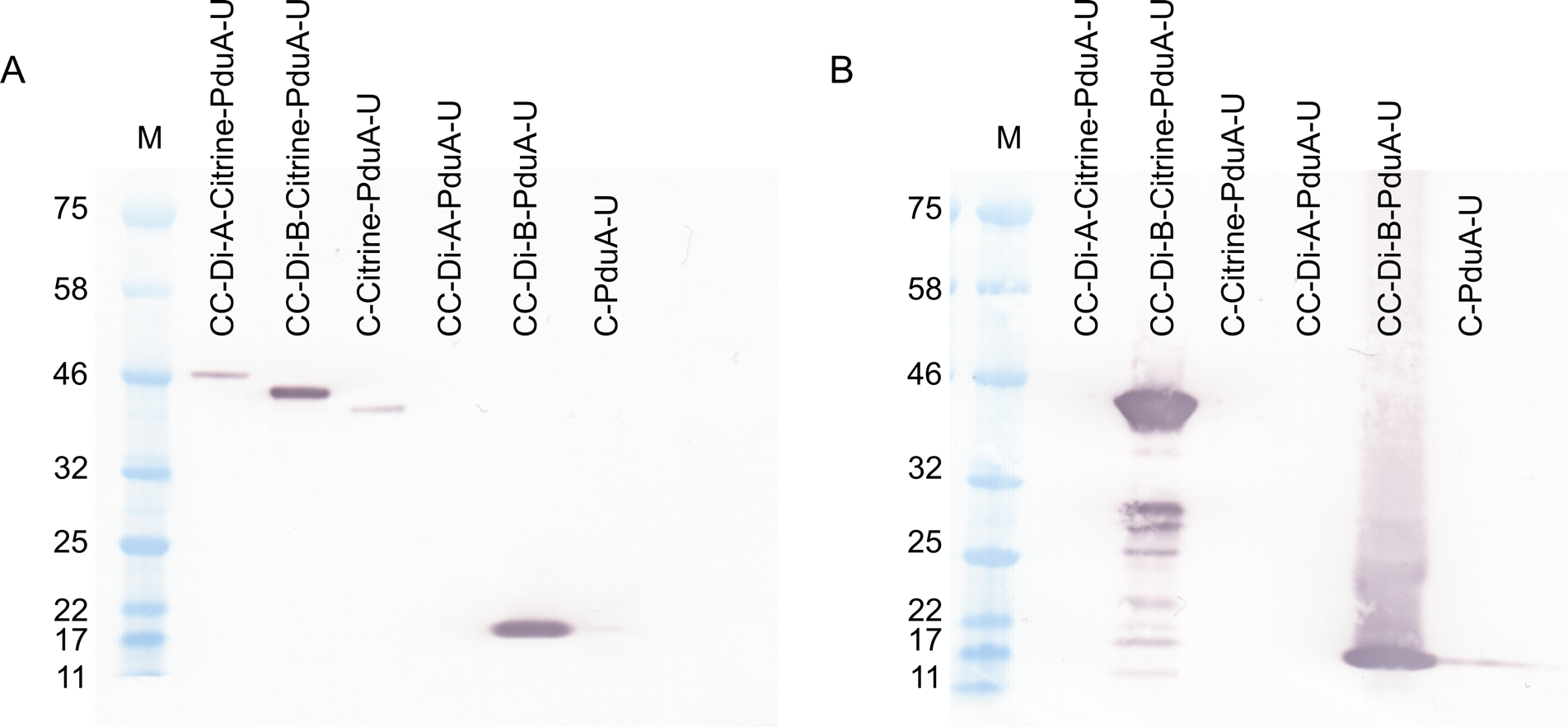
**Supplementary Figure 4. Western Blot analysis of coiled-coil tagged BMCs in comparison to a molecular weight marker (M) (kDa).** Samples were analyzed by SDS-PAGE and subsequently western blotted with an anti-His primary antibody, designed to bind to the His-tag present on all of the PduA fusions. (**A**) Lysates from a 1 g wet cell pellet and (**B**) final BMC containing purified fractions in comparison to a molecular weight standard.


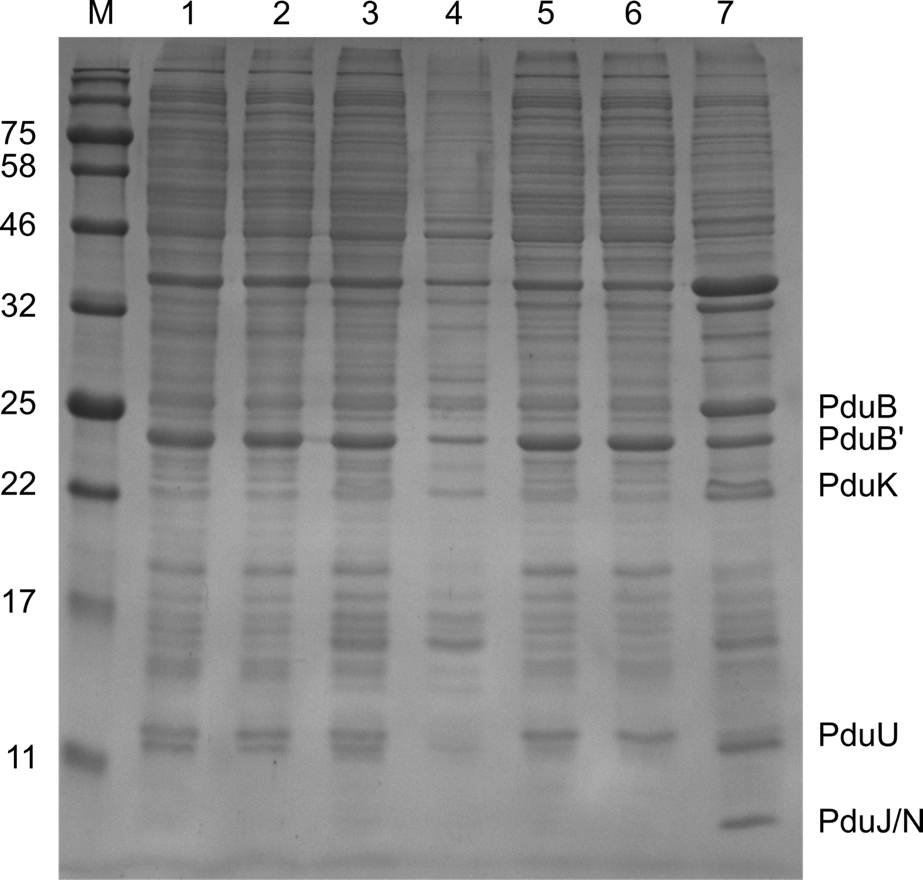


**Supplementary Figure 5.** **SDS-PAGE analysis of CC-Di-A labeled BMC purification in comparison to a molecular weight marker (M) (kDa).** Lane 1 – lysate, lane 2 – supernatant after centrifugation at 11,300 x g, lane 3 – pellet after centrifugation (resuspended in 20 mM Tris-HCl, pH 8.0, containing 20 mM NaCl), lane 4 – pellet after centrifugation at 11,000 x g (resuspended in 20 mM Tris-HCl, pH 8.0, containing 20 mM NaCl), lane 5 – supernatant after centrifugation, lane 6 - supernatant after centrifugation in 80 mM NaCl, lane 7 – pellet after centrifugation in 80 mM NaCl, resuspended in 20 mM Tris-HCl pH 8. Sample volumes of 2 µL were loaded in lanes 1 and 2, whilst lanes 3 to 7 were loaded with 5 μL sample.


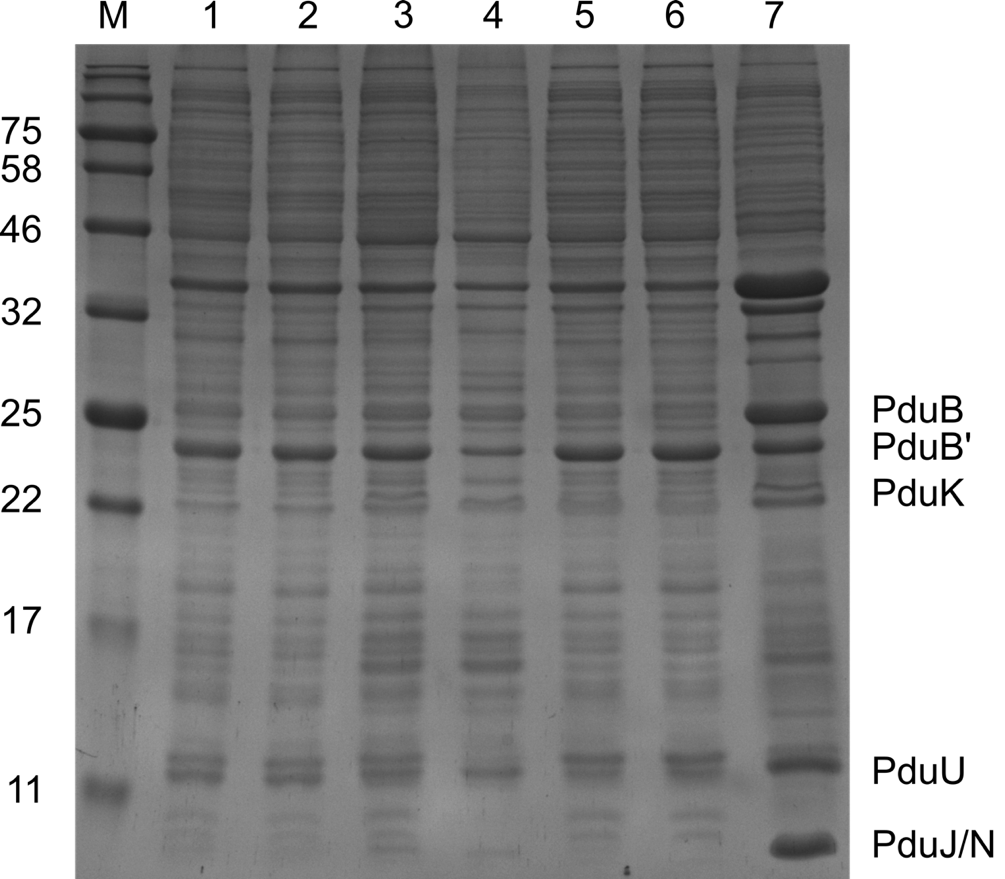

**Supplementary Figure 6.** **SDS-PAGE analysis of C labeled BMC purification in comparison to a molecular weight marker (M) (kDa).** Lane 1 – lysate, lane 2 – supernatant after centrifugation at 11,300 x g, lane 3 – pellet after centrifugation (resuspended in 20 mM Tris-HCl, pH 8.0, containing 20 mM NaCl), lane 4 – pellet after centrifugation at 11,000 x g (resuspended in 20 mM Tris-HCl, pH 8.0, containing 20 mM NaCl), lane 5 – supernatant after centrifugation, lane 6 - supernatant after centrifugation in 80 mM NaCl, lane 7 – pellet after centrifugation in 80 mM NaCl, resuspended in 20 mM Tris-HCl pH 8. Sample volumes of 2 µL were loaded in lanes 1 and 2, whilst lanes 3 to 7 were loaded with 5 μL sample.


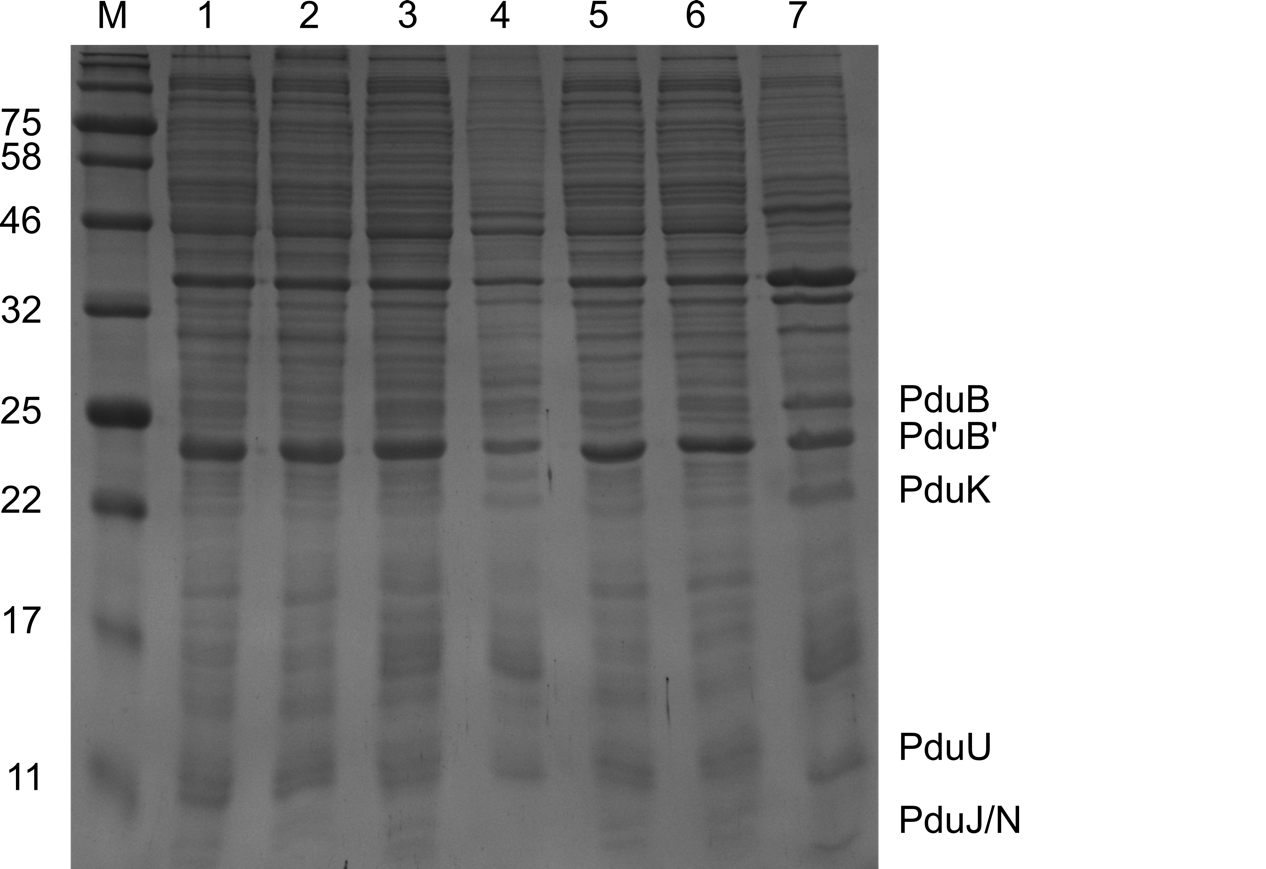


**Supplementary Figure 7.** **SDS-PAGE analysis of CC-Di-A-Citrine labeled BMC purification in comparison to a molecular weight marker (M) (kDa).** Lane 1 – lysate, lane 2 – supernatant after centrifugation at 11,300 x g, lane 3 – pellet after centrifugation (resuspended in 20 mM Tris-HCl, pH 8.0, containing 20 mM NaCl), lane 4 – pellet after centrifugation at 11,000 x g (resuspended in 20 mM Tris-HCl, pH 8.0, containing 20 mM NaCl), lane 5 – supernatant after centrifugation, lane 6 - supernatant after centrifugation in 80 mM NaCl, lane 7 – pellet after centrifugation in 80 mM NaCl, resuspended in 20 mM Tris-HCl pH 8. Sample volumes of 2 µL were loaded in lanes 1 and 2, whilst lanes 3 to 7 were loaded with 5 μL sample.


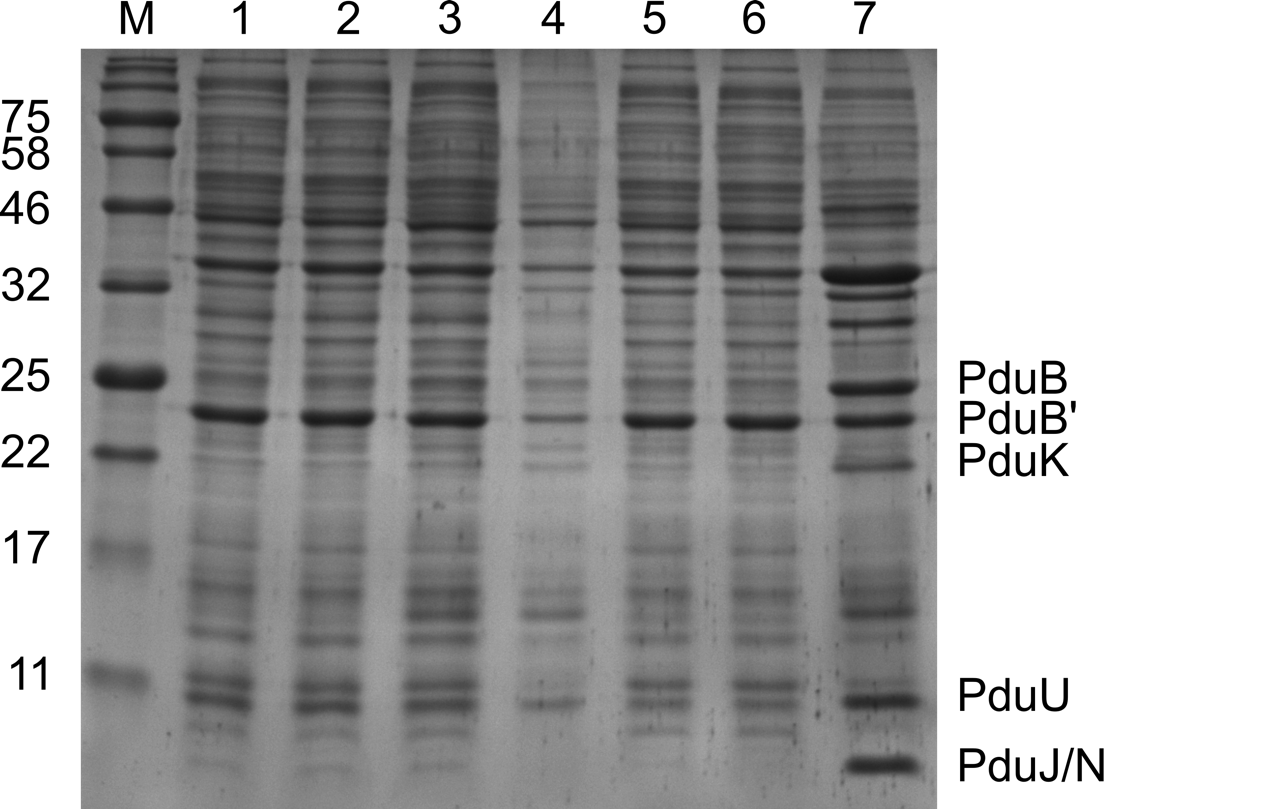


**Supplementary Figure 8.** **SDS-PAGE analysis of C-Citrine labeled BMC purification in comparison to a molecular weight marker (M) (kDa).** Lane 1 – lysate, lane 2 – supernatant after centrifugation at 11,300 x g, lane 3 – pellet after centrifugation (resuspended in 20 mM Tris-HCl, pH 8.0, containing 20 mM NaCl), lane 4 – pellet after centrifugation at 11,000 x g (resuspended in 20 mM Tris-HCl, pH 8.0, containing 20 mM NaCl), lane 5 – supernatant after centrifugation, lane 6 - supernatant after centrifugation in 80 mM NaCl, lane 7 – pellet after centrifugation in 80 mM NaCl, resuspended in 20 mM Tris-HCl, pH 8. Sample volumes of 2 µL were loaded in lanes 1 and 2, whilst lanes 3 to 7 were loaded with 5 μL sample.


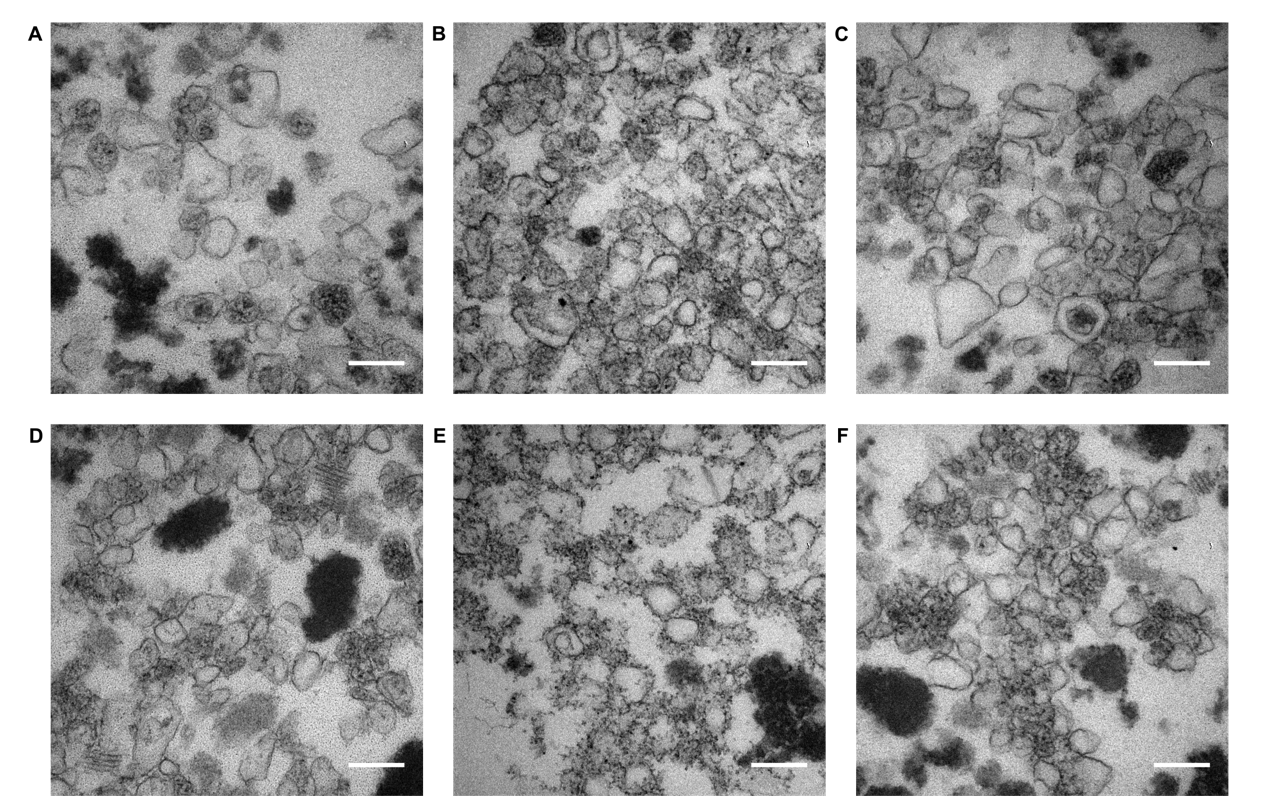


**Supplementary Figure 9.** **TEM analysis of resin embedded, thin-sectioned, purified BMCs.** BMCs extracted from *E. coli* BL21 * (DE3) cells expressing the following modified PduA proteins. (**A**) CC-Di-A-PduA-U. (**B**) CC-Di-B-PduA-U. (**C**) C-PduA-U. (**D**) CC-Di-A-Citrine-PduA-U. (**E**) CC-Di-B-Citrine-PduA-U. (**F**) C-Citrine-PduA-U. Scale bars show 0.2 µm.


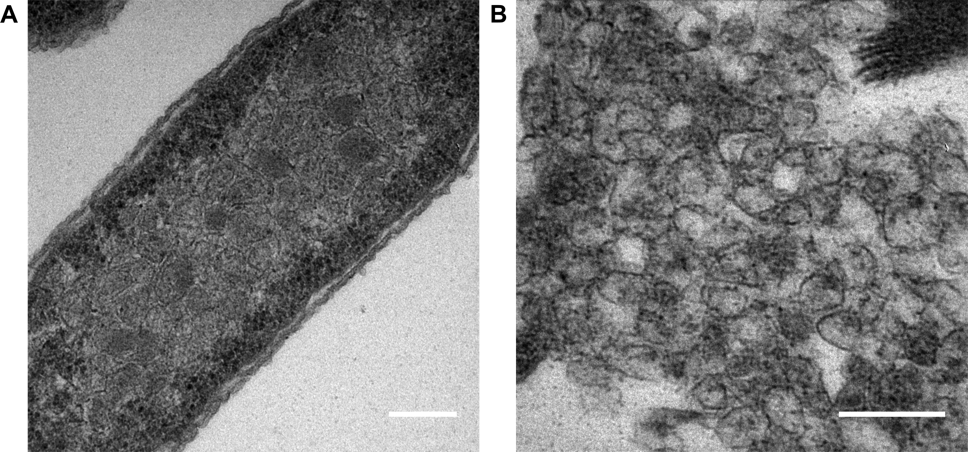


**Supplementary Figure 10.** **Recombinant production of empty BMCs.** TEM micrographs of *in-vivo* (**A**) and purified (**B**) empty Pdu BMCs formed from the shell proteins PduA, B, B’, J, K, N and U (PduA-U). Scale bar shows 0.2 µm.


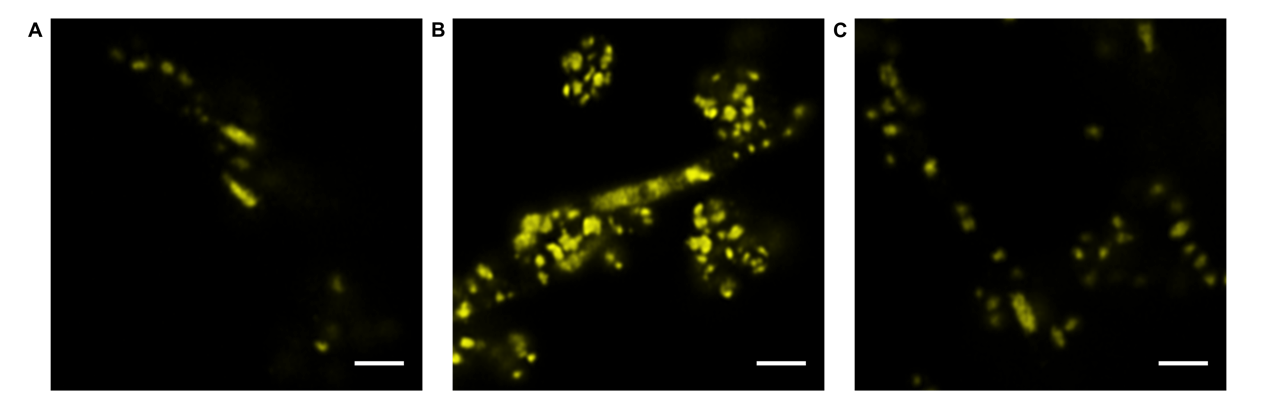


**Supplementary Figure 11.** **Expression of fluorescent coiled-coil labelled BMCs.** Confocal analysis of *E. coli* BL21 * (DE3) cells expressing (**A**) CC-Di-A-Citrine-PduA-U (**B**) CC-Di-B-Citrine-PduA-U (**C**) C-Citrine-PduA-U. Scale bars show 2 µm.


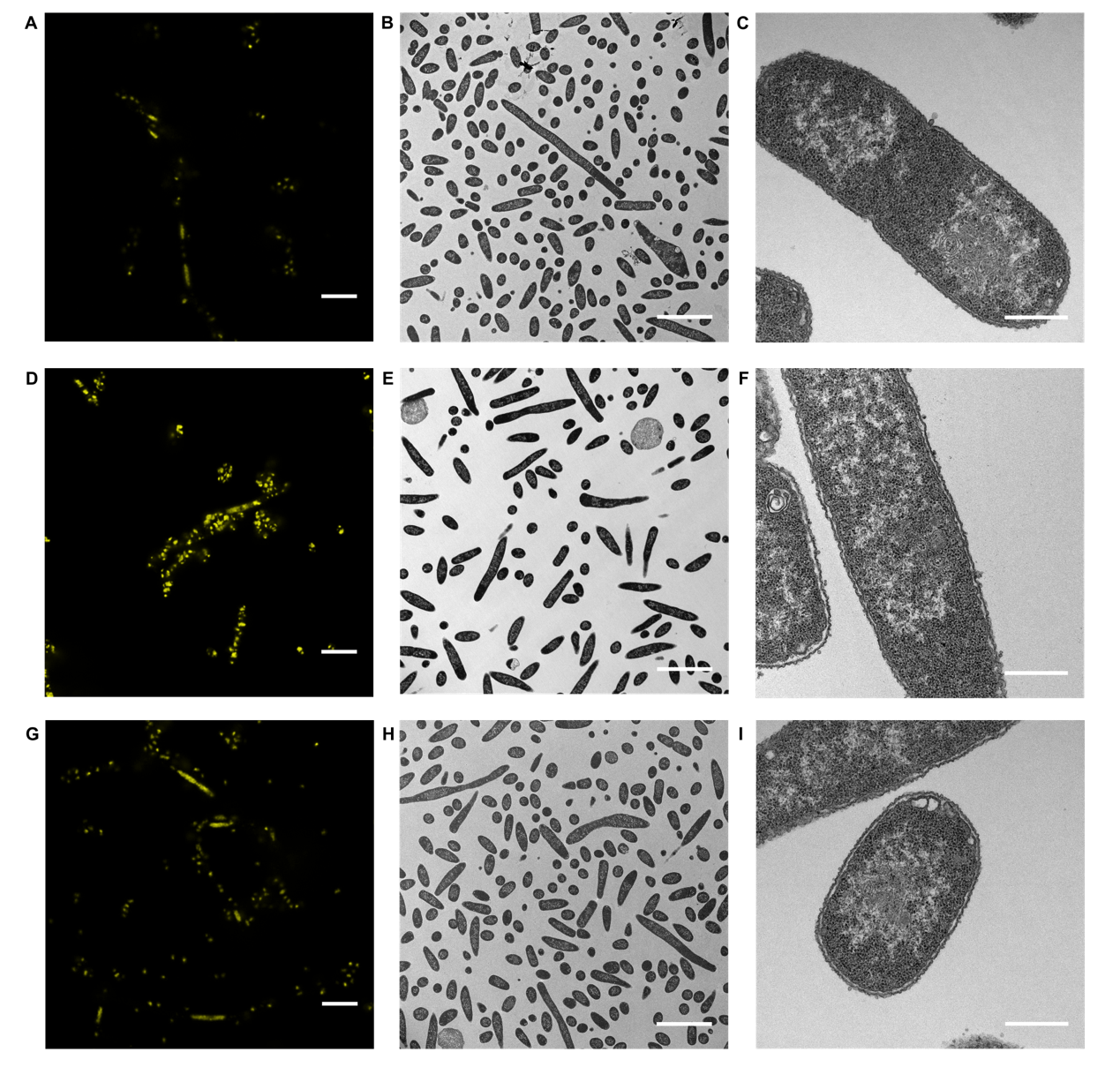

**Supplementary Figure 12.** **Expression of fluorescent coiled-coil labelled BMCs.** Full frame confocal and TEM analysis of *E. coli* BL21 * (DE3) cells after 2 hours of induction expressing (**A, B, C**) CC-Di-A-Citrine-PduA-U (**D, E, F**) CC-Di-B-Citrine-PduA-U (**G, H, I**) C-Citrine-PduA-U. Scale bars show 5 µm (A, B, D, E, G, H) and 0.5 µm (C, F, I).


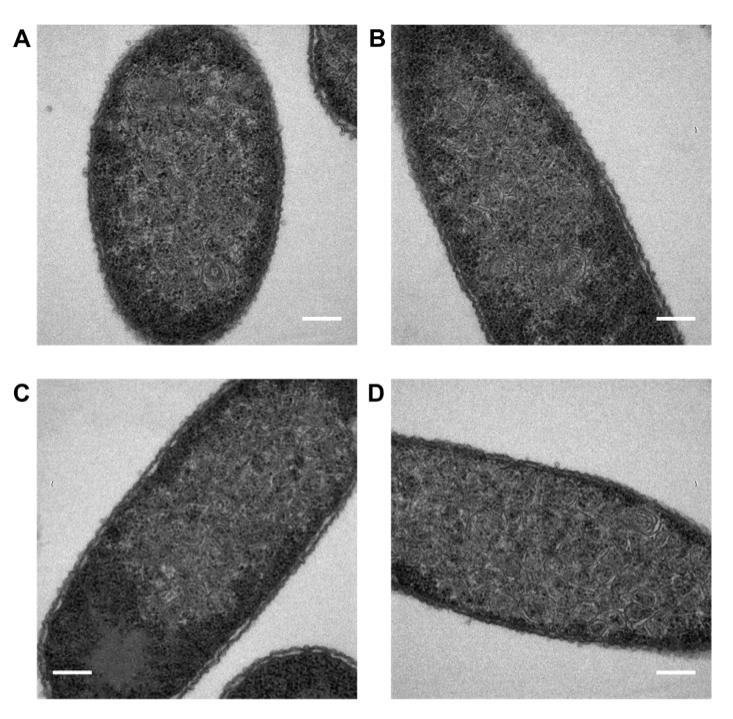


**Supplementary Figure 13.** **Minimal requirements for BMC formation.** TEM analyses of resin embedded, thin sectioned, *E. coli* BL21 * (DE3) cells producing the shell proteins PduB, B’, J, K, N and U (PduB-U). Scale bars show 0.2 µm


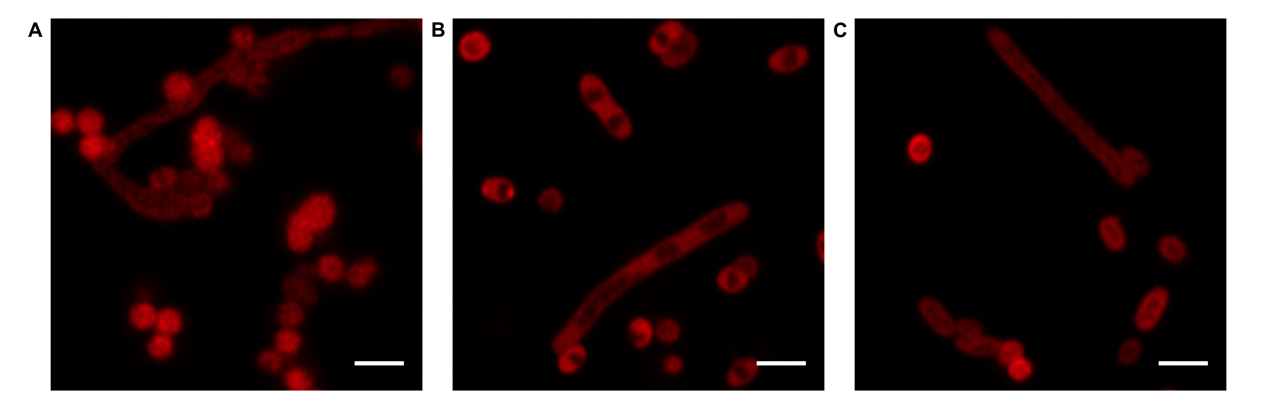


**Supplementary Figure 14.** **Expression of coiled-coil tagged mCherry.** Confocal analysis of *E. coli* BL21 * (DE3) cells expressing (**A**) CC-Di-A-mCherry (**B**) CC-Di-B-mCherry (**C**) C-mCherry. Scale bars show 2 µm.


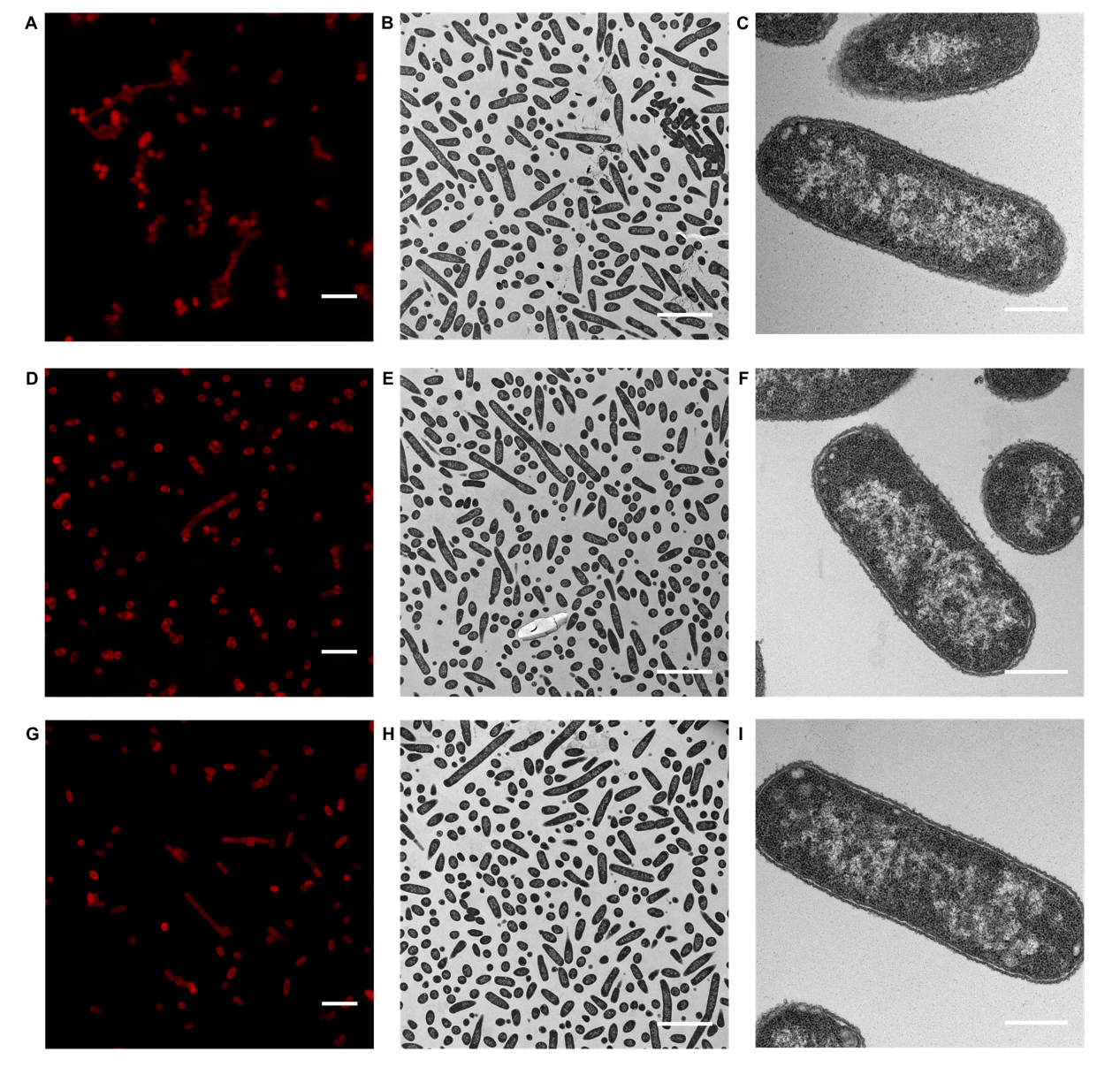

**Supplementary Figure 15.** **Expression of coiled-coil tagged mCherry.** Full frame confocal and TEM analysis of *E. coli* BL21 * (DE3) cells after 2 hours of induction expressing (**A, B, C**) CC-Di-A-mCherry (**D, E, F**) CC-Di-B-mCherry (**G, H, I**) C-mCherry. Scale bars show 5 µm (A, B, D, E, G, H) and 0.5 µm (C, F, I).


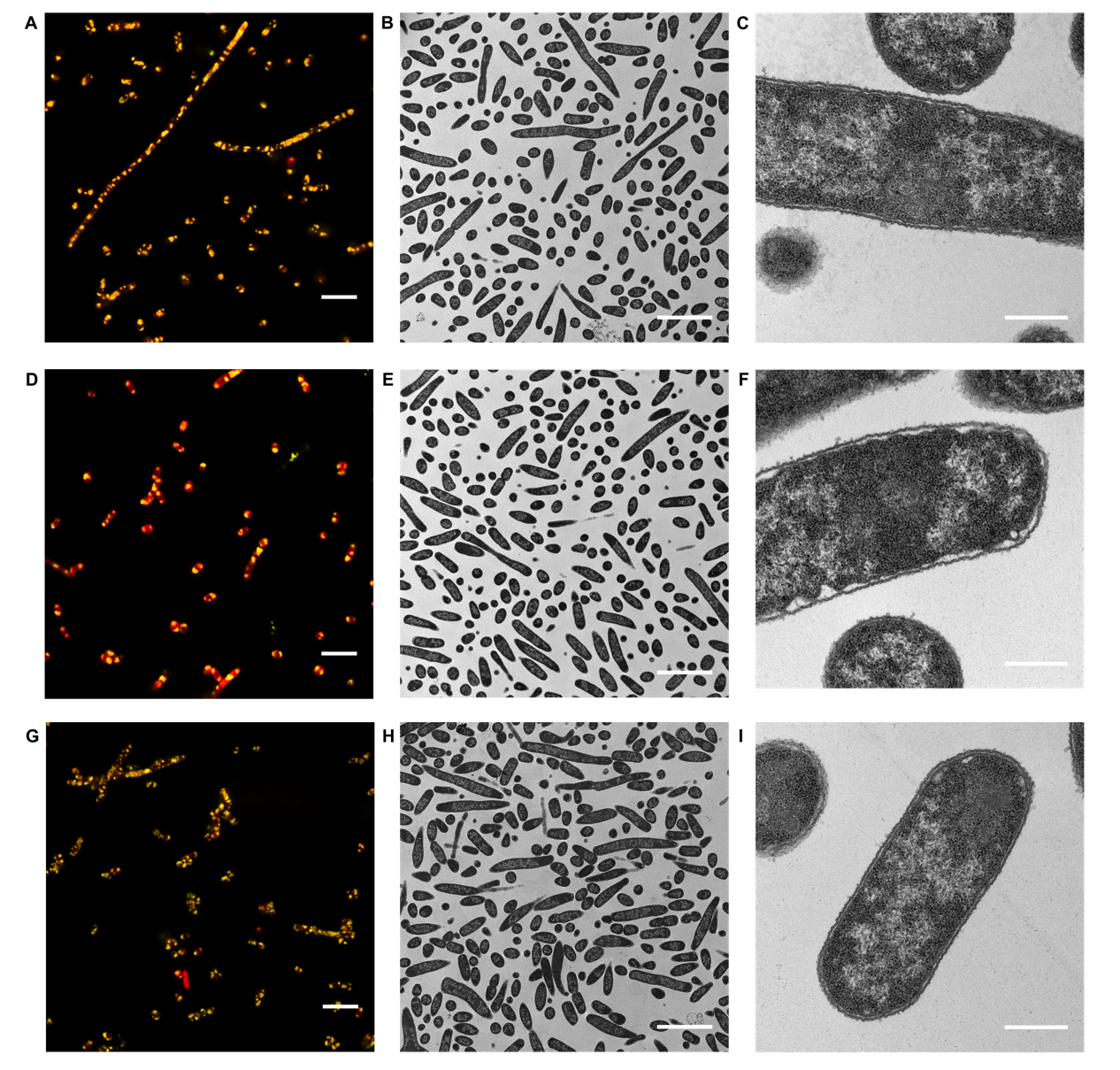

**Supplementary Figure 16.** **Localization of fluorescent proteins to coiled-coil labelled BMCs.** Full frame confocal and TEM analysis of *E. coli* BL21 * (DE3) cells after 2 hours of induction expressing CC-Di-B-Citrine-PduA-U with (**A, B, C**) CC-Di-A-mCherry (**D, E, F**) CC-Di-B-mCherry (**G, H, I**) C-mCherry. Scale bars show 5 µm (A, B, D, E, G, H) and 0.5 µm (C, F, I).


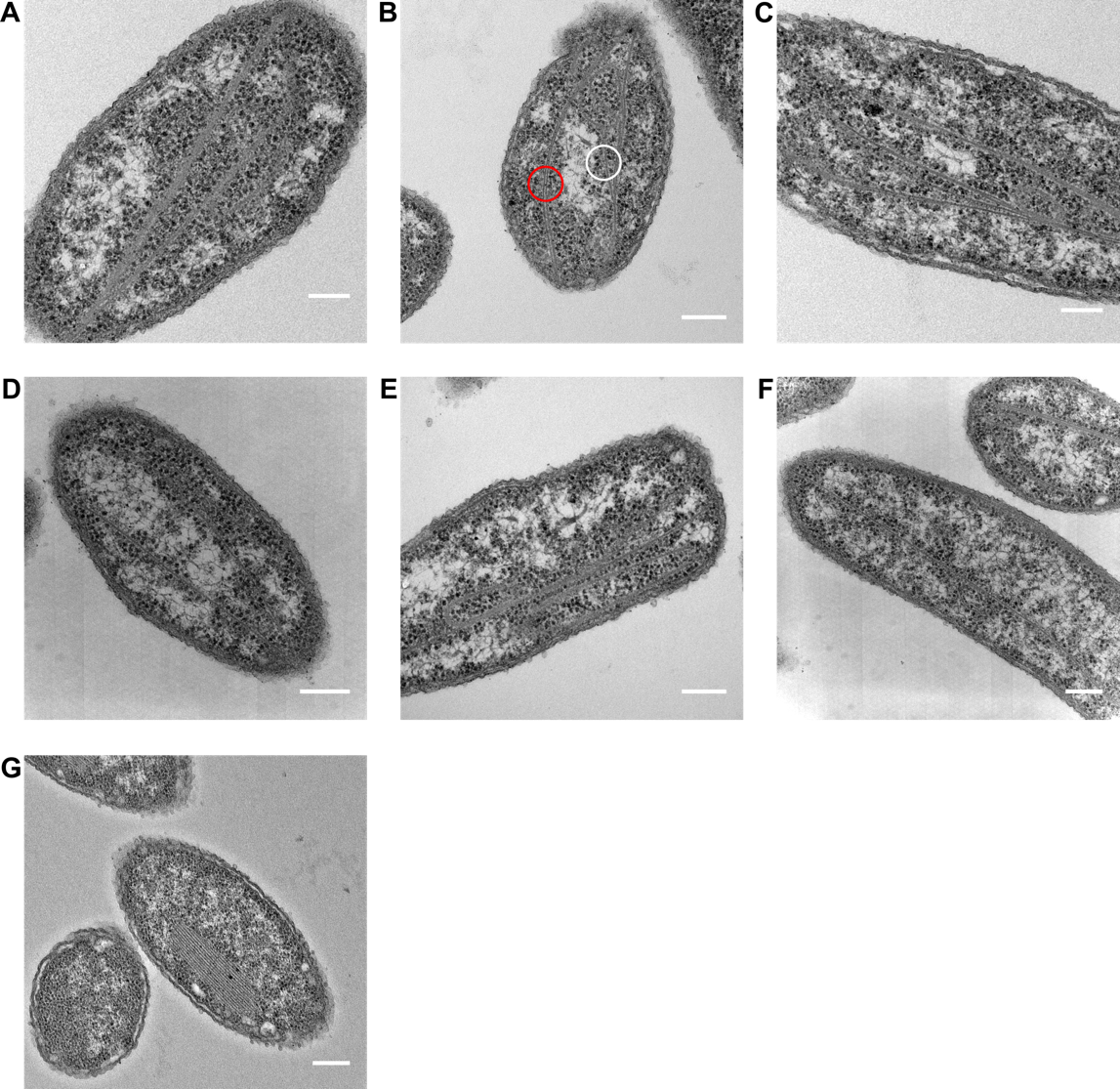


**Supplementary Figure 17. Expression of PduA variants in *E. coli*.** TEM analyses of resin embedded, thin sectioned, *E. coli* BL21 * (DE3) cells producing (**A – F**) a permuted variant of PduA (PduA^P^) and (**G**) unpermuted PduA. A single sheet is highlighted in (**B)** with a white circle, whereas two parallel sheets are highlighted with a red circle in the same panel. Scale bars show 0.2 µm.


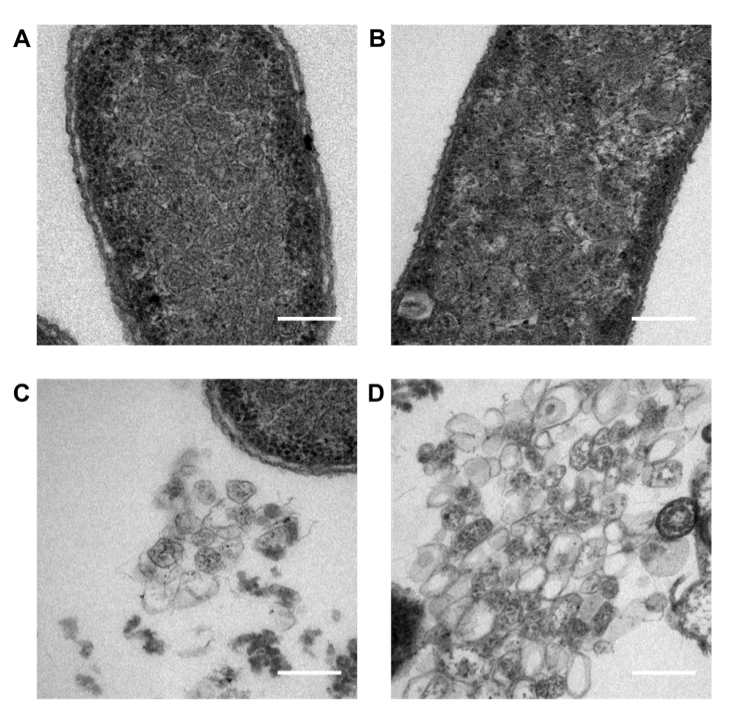


**Supplementary Figure 18.** **Expression of circularly permuted BMCs.** TEM analyses of resin embedded, thin sectioned, *E. coli* BL21 * (DE3) cells producing wild type PduA BMCs and circularly permuted PduA^P^ BMCs. (**A + C**) PduA-U *in-vivo* and ‘isolated’ (**B + D**) PduA^P^-U. Scale bars show 0.2 µm


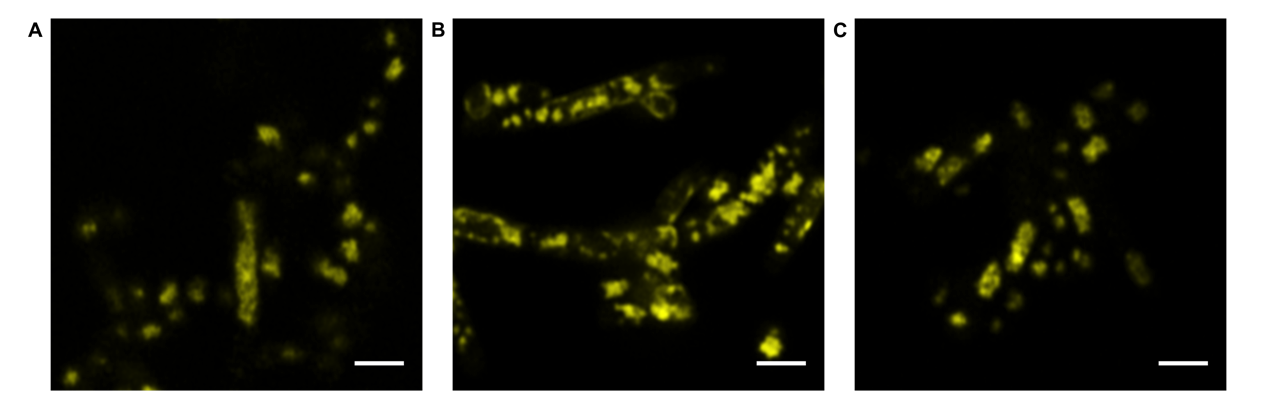
**Supplementary Figure 19.** **Expression of fluorescent, coiled-coil labelled, circularly permuted BMCs.** Confocal analysis of *E. coli* BL21 * (DE3) cells expressing (**A**) CC-Di-A-Citrine-PduA^P^-U (**B**) CC-Di-B-Citrine-PduA^P^-U (**C**) C-Citrine-PduA^P^-U. Scale bars show 2 µm.


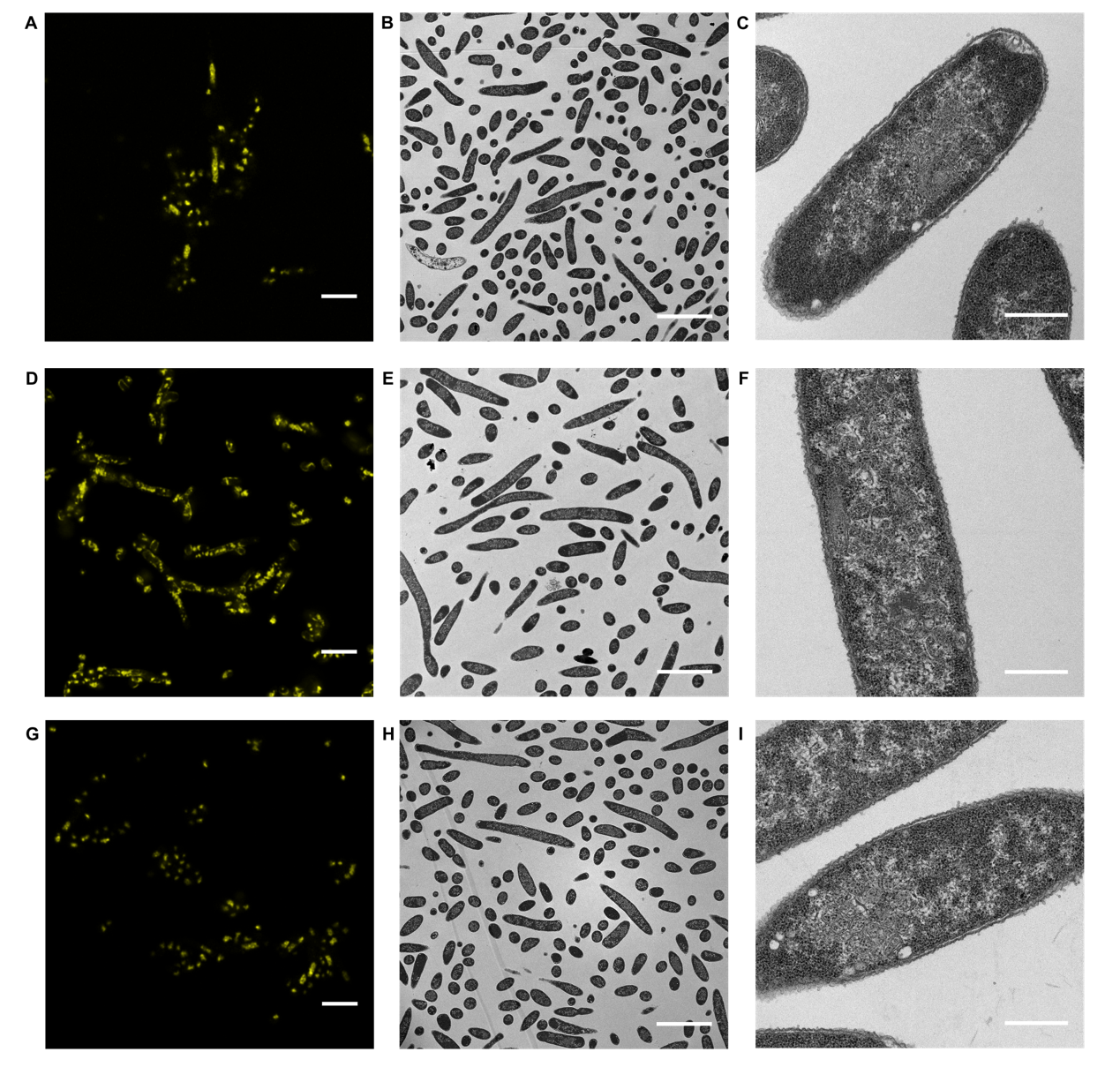

**Supplementary Figure 20. Expression of fluorescent, coiled-coil labelled, circularly permuted BMCs.** Full frame confocal and TEM analysis of *E. coli* BL21 * (DE3) cells after 2 hours of induction expressing (**A, B, C**) CC-Di-A-Citrine-PduA^P^-U (**D, E, F**) CC-Di-B-Citrine-PduA^P^-U (**G, H, I**) C -Citrine-PduA^P^-U. Scale bars show 5 µm (A, B, D, E, G, H) and 0.5 µm (C, F, I).


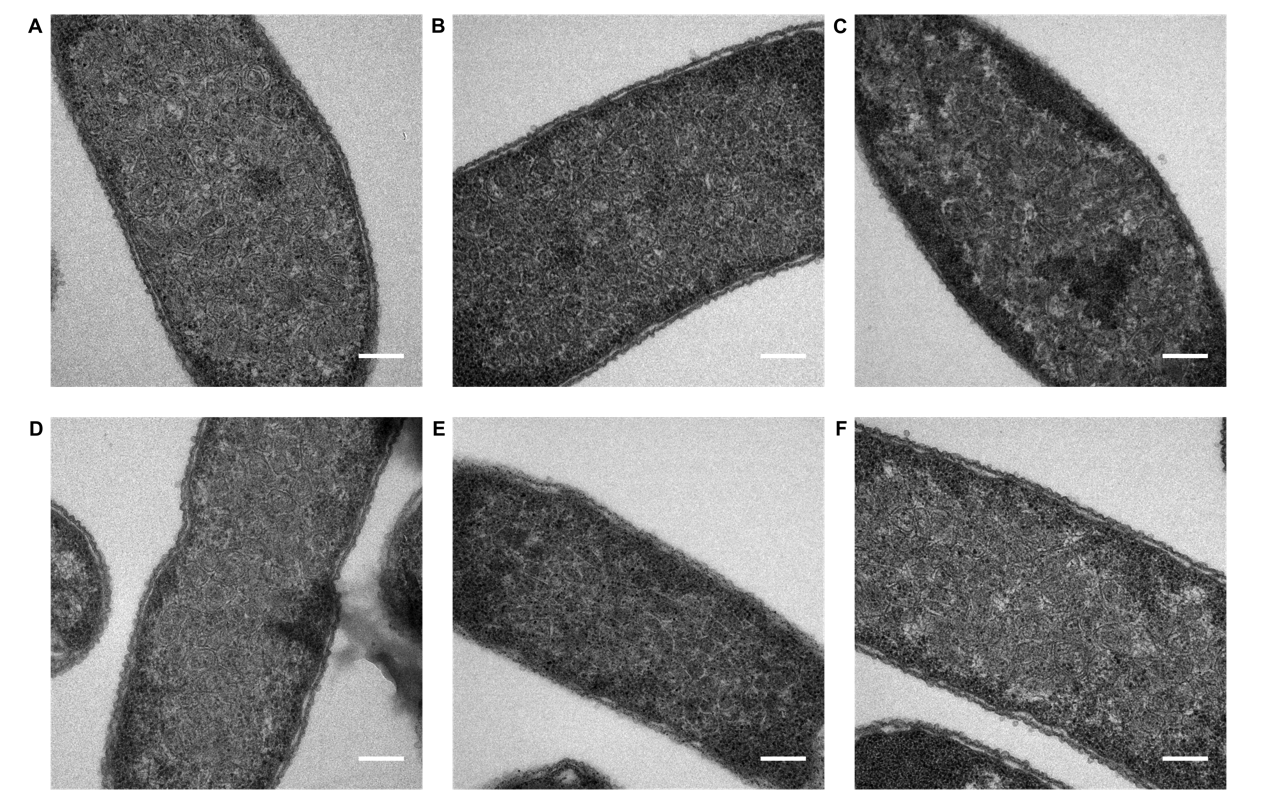


**Supplementary Figure 21.** ***In-vivo* expression of circularly permuted, coiled-coil labelled BMCs.** TEM analysis of *E. coli* BL21 * (DE3) cells expressing permuted PduA variants: (**A**) CC-Di-A-PduA^P^-U. (**B**) CC-Di-B-PduA^P^-U. (**C**) C-PduA^P^-U. **(D**) CC-Di-A-Citrine-PduA^P^-U. (**E**) CC-Di-B-Citrine-PduA^P^-U. (**F**) C-Citrine-PduA^P^-U. Scale bars show 0.2 µm.


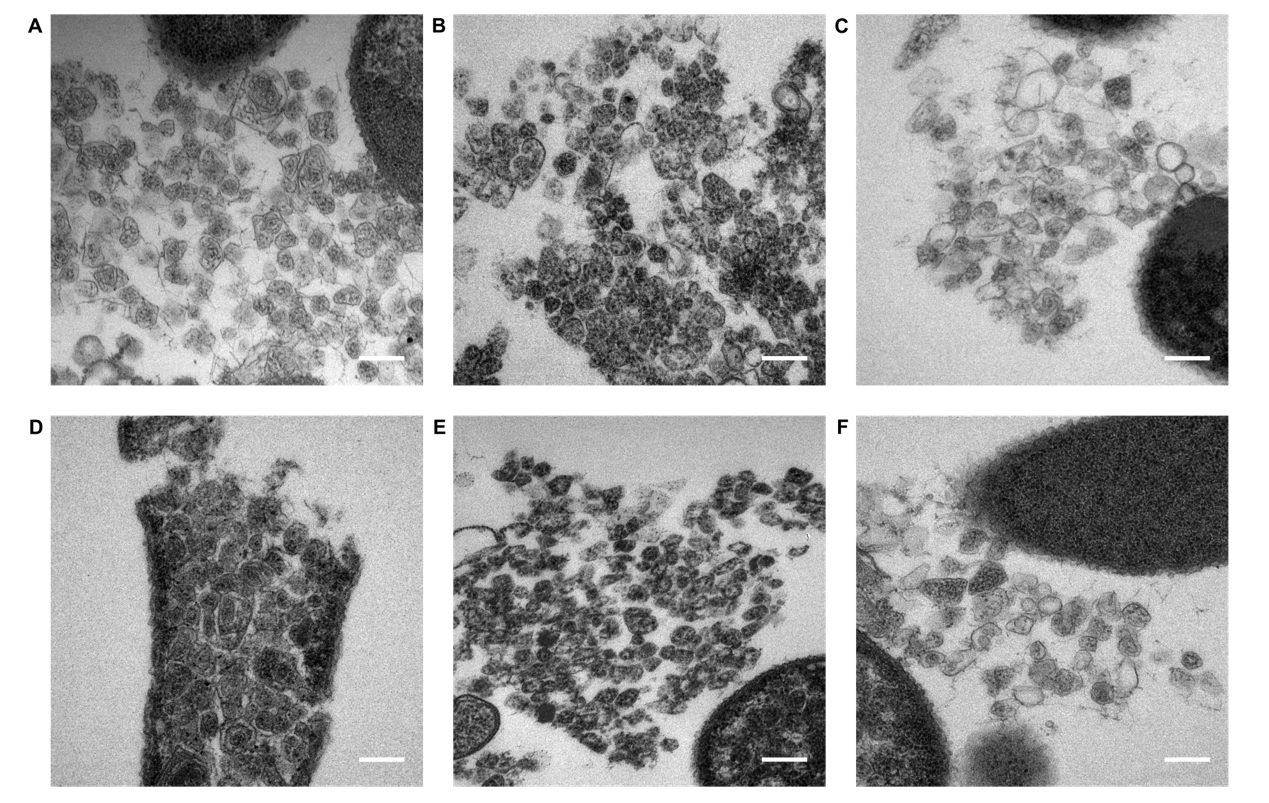


**Supplementary Figure 22. TEM analysis of coiled-coil tagged circularly permuted BMCs.** TEM analysis of resin embedded, thin-sectioned, permuted PduA BMCs tagged with coiled-peptides from lysed *E. coli* BL21 * (DE3) cells expressing the following modified PduA proteins. (**A**) CC-Di-A-PduA^P^-U. (**B**) CC-Di-B-PduA^P^-U. (**C**) C-PduA^P^-U. (**D**) CC-Di-A-Citrine-PduA^P^-U. (**E**) CC-Di-B-Citrine-PduA^P^-U. (**F**) C-Citrine-PduA^P^-U. Scale bars show 0.2 µm.


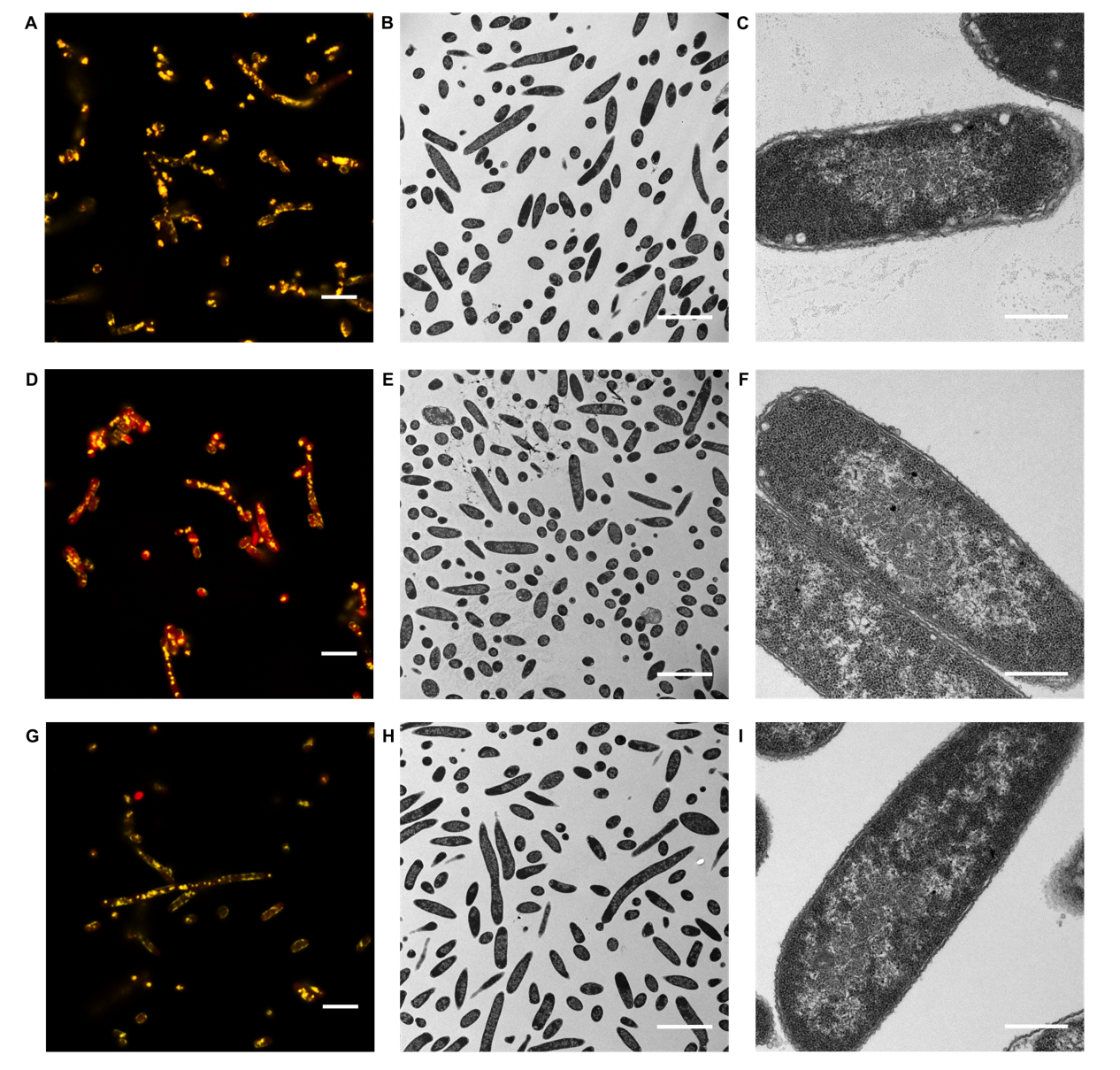

**Supplementary Figure 23.** **Localization of fluorescent proteins to circularly permuted coiled-coil labelled BMCs.** Full frame confocal and TEM analysis of *E. coli* BL21 * (DE3) cells after 2 hours of induction expressing CC-Di-B-PduA^P^-U with (**A, B, C**) CC-Di-A-mCherry (**D, E, F**) CC-Di-B-mCherry (**G, H, I**) C-mCherry. Scale bars show 5 µm (A, B, D, E, G, H) and 0.5 µm (C, F, I).

**Supplementary Table 1.** **Description of the fusion proteins constructed for this study**. CC-Di-A / CC-Di-B – *de novo* designed coiled-coil peptides, C – Control lacking a coiled-coil peptide, (His)_6_ – hexahistidine tag, TCS – thrombin cleavage sequence, Citrine – improved variant of yellow fluorescent protein, PduA – native *Citrobacter freundii* PduA, PduA^P^ – circularly permuted PduA, mCherry – red fluorescent protein variant.

| **Protein Name** | **Description** |
| --- | --- |
| CC-Di-A-PduA | CC-Di-A-(His)_6_-TCS-PduA |
| CC-Di-B-PduA | CC-Di-B-(His)_6_-TCS-PduA |
| C-PduA | C-(His)_6_-TCS-PduA |
| CC-Di-A-Citrine-PduA | CC-Di-A-(His)_6_-TCS-Citrine-PduA |
| CC-Di-B-Citrine-PduA | CC-Di-B-(His)_6_-TCS-Citrine-PduA |
| C-Citrine-PduA | C-(His)_6_-TCS-Citrine-PduA |
| CC-Di-A-PduA^P^ | CC-Di-A-(His)_6_-TCS-PduA^P^ |
| CC-Di-B-PduA^P^ | CC-Di-B-(His)_6_-TCS-PduA^P^ |
| C-PduA^P^ | C-(His)_6_-TCS-PduA^P^ |
| CC-Di-A-Citrine-PduA^P^ | CC-Di-A-(His)_6_-TCS-Citrine-PduA^P^ |
| CC-Di-B-Citrine-PduA^P^ | CC-Di-B-(His)_6_-TCS-Citrine-PduA^P^ |
| C-Citrine-PduA^P^ | C-(His)_6_-TCS-Citrine-PduA^P^ |
| CC-Di-A-mCherry | CC-Di-A-(His)_6_-TCS-mCherry |
| CC-Di-B-mCherry | CC-Di-B-(His)_6_-TCS-mCherry |
| C-mCherry | C-(His)_6_-TCS-mCherry |

**Supplementary Table 2.** **Oligonucleotides used in this study.** Restriction sites are underlined.

| **Name** | **Sequence 5’ – 3’** |
| --- | --- |
| Citrine AseI_FW | GACATTAATATGGTGAGCAAGGGCGAGGAGCTG |
| Citrine_SpeI_NdeI_RV | GACACTAGTAAACATATGCTTGTACAGCTCGTCCATGCCGAG |
| Citrine_NdeI_FW | CACCATATGGTGAGCAAGGGCGAGGAGC |
| Citrine_SpeI_RV | CACACTAGTTTACTTGTACAGCTCGTCC |
| mCherry_NdeI_FW | CAGCATATGGTGAGCAAGGGCGAGG |
| mCherry_SpeI_RV | GACACTAGTTTACTTGTACAGCTCGTCCATGC |

**Supplementary Table 3. Strains used in this study.**

| **Strain** | **Genotype** | **Source** |
| --- | --- | --- |
| JM109 | endA1, recA1, gyrA96, thi, hsdR17 (rk–, mk+), relA1, supE44, Δ(lac-proAB), [F′, traD36, proAB, laqIqZΔM15] | Promega |
| BL21 * (DE3) | F– ompT hsdSB (rB– mB–) gal dcm (DE3) | Novagen |
