# Supplementary material for: De novo targeting to the cytoplasmic and luminal side of bacterial microcompartments
Source: Nat Commun. 2018 Aug 24;9:3413. doi: 10.1038/s41467-018-05922-x (PMC6109187; doi:10.1038/s41467-018-05922-x)
Supplement: Supplementary file 2 — Descriptions of Additional Supplementary Files [file 41467_2018_5922_MOESM2_ESM.pdf]

Descriptions of Additional Supplementary Files

File Name: Supplementary Movie 1
Description: TEM tomography of E. coli BL21 * (DE3) cells producing a permuted variant of PduA (PduAP)

File Name: Supplementary Dataset 1
Description: Synthesized DNA – cloned into XbaI/NdeI sites of pET14b

File Name: Supplementary Dataset 2
Description: Synthesized permuted PduA – cloned into NdeI/BamHI sites of pET3a

File Name: Supplementary Dataset 3
Description: N-Terminal coiled-coil peptides, coiled-coil regions underlined

File Name: Supplementary Dataset 4
Description: Plasmids Used in this study
